# Supplementary material for: Cell-DINO: Self-supervised image-based embeddings for cell fluorescent microscopy
Source: PLoS Comput Biol. 2025 Dec 29;21(12):e1013828. doi: 10.1371/journal.pcbi.1013828 (PMC12826486; doi:10.1371/journal.pcbi.1013828)
Supplement: S1 Text — (PDF) [file pcbi.1013828.s001.pdf]

Cell-DINO: Self-Supervised Image-based Embeddings for Cell Fluorescent Microscopy: Supplementary Material

Recommendations for adopting Cell-DINO

Cell-DINO requires GPU acceleration, which are becoming more accessible to labs around the world as AI infrastructure keeps growing in academia and government. We encourage scientists to experiment with Cell-DINO in the following way:

1. First, explore pre-trained Cell-DINO models in the specific application of interest. The models can be used out-of-the-box for feature extraction if the number of channels matches. If not, explore the next options.

2. If the number of channels do not match, explore our recently released DINO-BOC model [4], which is channel-agnostic. It has been trained with the same principles of Cell-DINO to process one channel at a time.

3. Design a benchmark to measure success. This is highly specific to the application of interest, and may or may not require labels.

4. After running the benchmark with Cell-DINO embeddings, compare against simpler approaches as a sanity check. For instance, use CellProfiler features or other models released by the community.

5. If the results are not satisfactory, explore training your own Cell-DINO model. Start training a ViT-small model and continue benchmarking to evaluate whether the new results are successful or not. Growing the model size may improve performance, but it also requires more resources or time to converge.

6. We recommend initializing weights with DINOv2 or Cell-DINO weights, as our experiments indicate that model initialization can improve performance. However, this is not required, and needs to be guided by the benchmark.

Cell-DINO can be helpful in many applications, but it also requires resources, time, and expertise. As the community makes progress with training, validating, and releasing more models, its usability and accessibility will increase over time.

UMAP comparison: supervised vs self-supervised

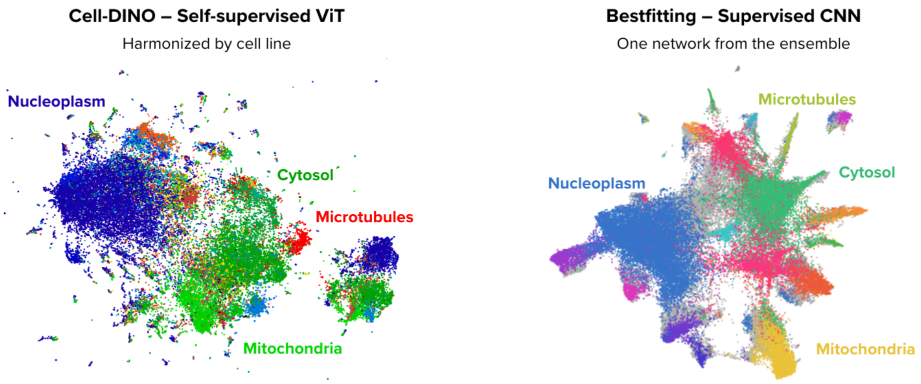

Figure A: **UMAP visualizations of image-based embeddings obtained on the HPA-FoV dataset.** The self-supervised approach Cell-DINO (left) finds clusters that resemble the clusters learned with supervised learning (right). Four major clusters are highlighted in each map: nucleoplasm, cytosol, microtubules, and mitochondria. The supervised approach was originally reported in [10] and the visualization is available in the Human Protein Atlas website through the following link: `https://www.proteinatlas.org/humanproteome/subcellular/location+umap` (accessed August 2025).

Cell-DINO embeddings encode technical variation

Batch effects is a broad term used to describe unwanted variation in biological measurements, which is observed in imaging as well as in many other data modalities [7, 5]. Batch effects in imaging are originated by complex combinations of experimental factors such as plate effects, well position effects, environmental, and other technical variations. All feature extraction methods are prone to capture batch effects, which can confound the downstream analysis when the biological signal is overpowered by unwanted variation. This problem has been extensively studied in computational biology [3] and also in imaging specifically [2]. Cell-DINO embeddings are not immune to batch effects and require proper post-hoc correction to enhance biological signal.

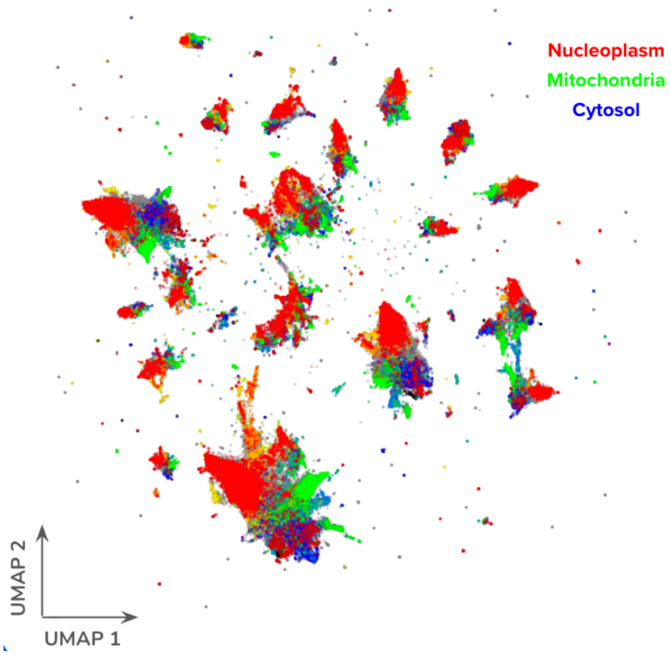

Figure B: **UMAP visualizations of image-based embeddings obtained on the HPA-FoV dataset before batch correction.** Each major cluster corresponds to one cell line. Note that protein localization labels are a secondary factor of variation, which can be recovered after data integration with batch correction methods (Figure. A)

Cell-DINO embeddings display signs of batch effects in both the HPA and the CPG datasets. This can be observed qualitatively in Figure CA for the HPA dataset, where some cell-line clusters exhibit fragmented subclusters in relatively distant neighborhoods. For example, the cell lines U251MG, U2OS, and A431 have three major well-aligned clusters, and three equally aligned minor clusters in the center of the plot. The fragmentation could be the result of batch effects, but to verify objectively, batch information is necessary (not fully available for the HPA Kaggle datasets used in this study). Nevertheless, we show how Cell-DINO embeddings can be transformed to reduce unwanted variation by using a popular batch correction algorithm from the single-cell genomics literature [7] to remove one prominent and known source of variation: cell line identity. The result in Figure CC shows that the transformation successfully integrates the data and reveals other biologically relevant information (protein localization) encoded in the Cell-DINO embeddings. This does not fully address batch effects in this example, but illustrates how Cell-DINO embeddings encode rich phenotypic information that can be recovered with appropriate transformations.

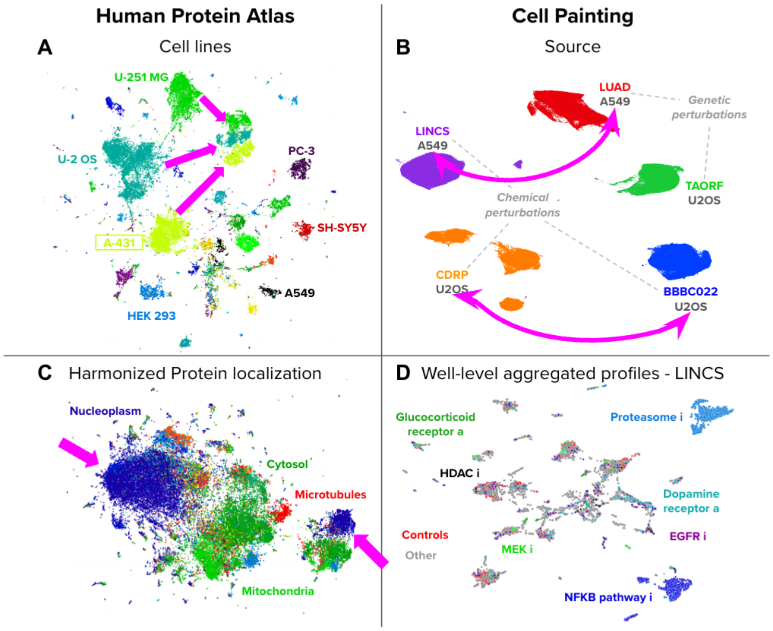

Figure C: **UMAP visualizations of image-based embeddings obtained with Cell-DINO.** Pink arrows indicate possible clustering due to batch effects. Left column: Image-based embeddings of the HPA-FoV dataset. Right column: Image-based embeddings of the Cell Painting datasets. Top row (A,B): unprocessed embeddings. Bottom row (C,D): transformed embeddings for downstream analysis.

The CPG datasets also exhibit major batch clusters with the unprocessed Cell-DINO embeddings. The UMAP visualization in Figure CB shows how single cells from the same cell line are appart because they belong to different datasets. Following a standard data analysis pipeline for Cell Painting, which includes aggregation and batch correction, the initial data is transformed into more coherent clusters that group treatments with similar mechanisms of action (Figure CD). The CPG datasets include extensive metadata with experimental details useful for addressing biases and correct measurements. The batch correction algorithm used in this case was sphering [1, 9], which uses control samples as a reference to remove nuisance variation from treatments. These examples show that Cell-DINO embeddings encode rich image features that include not only phenotypic information, but also technical variation. However, the embeddings are sufficiently disentangled to facilitate various types of image-based analyses with the use of post-processing transformations that can mitgate batch effects and highlight relevant biological signal. Quantitative results comparing the effects of not using batch correction are

reported in Table 3 in the main manuscript.

Protein localization heterogeneity

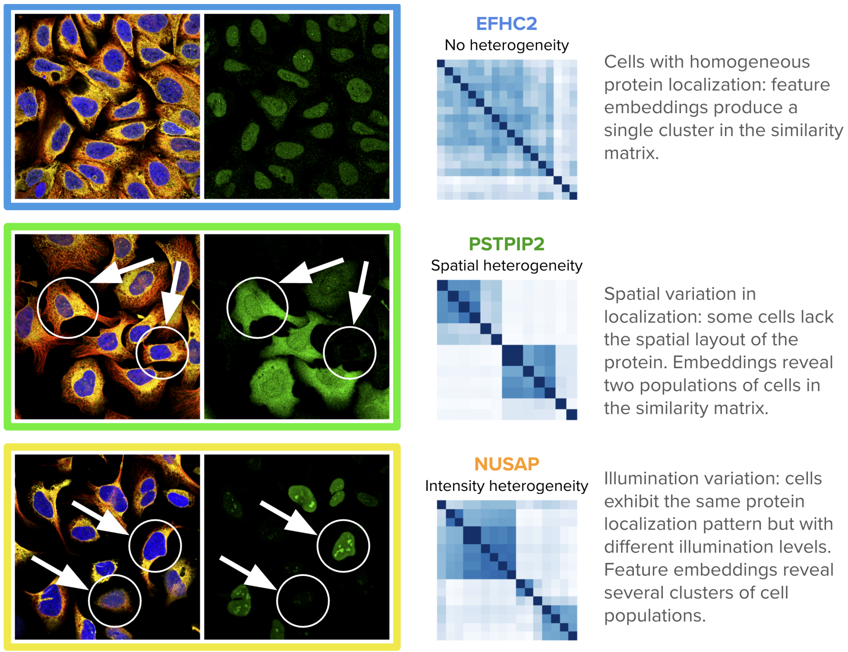

Figure D: **Large version of data in Figure 8 from main text.** Single-cell heterogeneity analysis on the HPA-SC dataset. Only U2OS cells were selected for this analysis to measure morphological differences for three proteins EFHC2, PSTPIP2, and NUSAP, which have been annotated as exhibiting no heterogeneity, spatial heterogeneity, and intensity heterogeneity, respectively. The matrices display similarity of single cells from images labeled for the corresponding proteins using Cell-DINO embeddings. Color images illustrate the composite multi-channel visualization, and green images represent the corresponding protein channel. The arrows point to cells that differ in their protein localization activity, explaining the heterogeneity patterns observed.

Further comparison with SubCell

To better assess the performance improvements of Cell-DINO compared to Subcell's models, we evaluate our method on the protein localization task of the HPAv23 dataset in a setting close to that described by Subcell's authors and report the results in tables A and B. In particular:

- We reproduce their data splits and data filtering processes.
- Instead of a linear layer, we train a multi-layer perceptron on top of frozen features with their exact specifications.
- We use their focal loss implementation instead of a cross-entropy loss.
- We report the Mean Average Precision of our experiments.
- To obtain FOV evaluations, we average the logits of single-cells comprising a given FOV as suggested in their supplementary material.
- We report both 19 classes and 31 classes evaluations to follow their experiments.

Table A: Cell-DINO compared with Subcell's baselines on the protein localization task of HPAv23 with the 19 classes used in the Kaggle competition.

Method	Single Cell (MAP)	FOV (MAP)
MAE-CellS-ProtS-Pool	60.0	67.4
ViT-ProtS-Pool	61.3	70.0
Cell-DINO	**66.4**	**71.4**

Table B: Cell-DINO compared with Subcell's baselines on the protein localization task of HPAv23 with 31 classes.

Method	Single Cell (MAP)	FOV (MAP)
MAE-CellS-ProtS-Pool	39.9	46.0
ViT-ProtS-Pool	42.1	51.2
Cell-DINO	**44.2**	**51.4**

We obtained these implementation details from Subcell's code-bases. In our experiments, using harmony did not significantly improve performance on this data.

Fine-tuning CellDINO on HPA-FOV

Only CellDINO+ fine-tuned results are shown in Figure 3 to inform on the best performance that could be achieved by combining DINOv2 pretraining and fine-tuning. For completeness, we also ran fine-tuning on Cell-DINO and report results

in Table C, as expected the results are much improved compared to Cell-DINO, but slightly worse than Cell-DINO+ fine-tuned.

Table C: Comparison of Cell-DINO fine-tuned and Cell-DINO+ fine-tuned on HPA-FoV in terms of F1 scores.

Task	Cell-DINO	Cell-DINO ft	Cell-DINO+	Cell-DINO+ ft
Cell line classification	83.3	90.6	90.0	91.2
Protein localization	65.0	81.9	67.8	83.7

LINCS Cell Painting evaluation with different doses

Table D: Dose effect evaluation. Comparison of model performance in the mechanism of action (MoA) classification task using the LINCS Cell Painting dataset. The reported values are the area under the precision-recall curve, following the evaluation protocol in [11].

Method	Low dose	Medium dose	Maximum dose
OpenPhenom [8]	4.97	5.92	6.40
CellProfiler	5.17	6.00	6.70
CP-CNN [9]	**5.58**	6.49	7.32
SubCell [6]	5.49	6.67	7.65
Cell-DINO	5.47	**6.70**	**7.67**

The results in Table D show how the ability of all models to distinguish MoAs from image-based profiles decreases with dose. The results are the average of ten runs for each model-dose combination. The phenotype of drugs activates at characteristic dosages, which are usually identified using drug-response curves. Our analysis is consistent with the idea that some phenotypes are either not activated or not strong enough to be detected with image analysis. However, our Cell-DINO approach remains robust and performs well as the dosage decreases, outperforming other methods at medium and maximum doses and remaining competitive at low doses.

References

[1] D Michael Ando, Cory Y McLean, and Marc Berndl. Improving phenotypic measurements in high-content imaging screens. *BioRxiv*, page 161422, 2017.

[2] John Arevalo, Ellen Su, Jessica D Ewald, Robert Van Dijk, Anne E Carpenter, and Shantanu Singh. Evaluating batch correction methods for image-based cell profiling. *Nature Communications*, 15(1):6516, 2024.

[3] Maren Büttner, Zhichao Miao, F Alexander Wolf, Sarah A Teichmann, and Fabian J Theis. A test metric for assessing single-cell rna-seq batch correction. *Nature methods*, 16(1):43–49, 2019.

[4] Alice V De Lorenci, Seung Eun Yi, Théo Moutakanni, Piotr Bojanowski, Camille Couprie, Juan C Caicedo, and Wolfgang Maximilian Anton Pernice. Scaling channel-adaptive self-supervised learning. *Transactions on Machine Learning Research*, 2025.

[5] Wilson Wen Bin Goh, Wei Wang, and Limsoon Wong. Why batch effects matter in omics data, and how to avoid them. *Trends in biotechnology*, 35(6):498–507, 2017.

[6] Ankit Gupta, Zoe Wefers, Konstantin Kahnert, Jan N Hansen, William D Leineweber, Anthony Cesnik, Dan Lu, Ulrika Axelsson, Frederic Balllosera Navarro, Theofanis Karaletsos, et al. Subcell: Vision foundation models for microscopy capture single-cell biology. *bioRxiv*, pages 2024–12, 2024.

[7] I. Korsunsky et al. Fast, sensitive and accurate integration of single-cell data with Harmony. *Nat. Methods*, 16, 2019.

[8] Oren Kraus, Kian Kenyon-Dean, Saber Saberian, Maryam Fallah, Peter McLean, Jess Leung, Vasudev Sharma, Ayla Khan, Jia Balakrishnan, Safiye Celik, et al. Masked autoencoders for microscopy are scalable learners of cellular biology. In *Proceedings of the IEEE/CVF Conference on Computer Vision and Pattern Recognition*, 2024.

[9] Nikita Moshkov, Michael Bornholdt, Santiago Benoit, Matthew Smith, Claire McQuin, Allen Goodman, Rebecca A Senft, Yu Han, Mehrtash Babadi, Peter Horvath, et al. Learning representations for image-based profiling of perturbations. *Nature communications*, 15(1):1594, 2024.

[10] Wei Ouyang, Casper F Winsnes, Martin Hjelmare, Anthony J Cesnik, Lovisa Åkesson, Hao Xu, Devin P Sullivan, Shubin Dai, Jun Lan, Park Jinmo, et al.

Analysis of the human protein atlas image classification competition. *Nature methods*, 16(12):1254–1261, 2019.

[11] G.P. Way, T. Natoli, A. Adeboye, L. Litichevskiy, A. Yang, X. Lu, J.C. Caicedo, B.A. Cimini, K. Karhohs, D.J. Logan, M.H. Rohban, M. Kost-Alimova, K. Hartland, M. Bornholdt, S.N. Chandrasekaran, M. Haghighi, E. Weisbart, S. Singh, A. Subramanian, and A.E. Carpenter. Morphology and gene expression profiling provide complementary information for mapping cell state. *Cell Systems*, 13:911–923, 2022.
