# Supplementary material for: Cell-DINO: Self-supervised image-based embeddings for cell fluorescent microscopy
Source: PLoS Comput Biol. 2025 Dec 29;21(12):e1013828. doi: 10.1371/journal.pcbi.1013828 (PMC12826486; doi:10.1371/journal.pcbi.1013828)
Supplement: Attachment — Submitted filename: responses.pdf [file pcbi.1013828.s002.pdf]

Cell-DINO: Self-Supervised Image-based Embeddings for Cell Fluorescent Microscopy: Response to reviews

Dear Editor,

Thank you very much for your invitation to submit a major revision of our manuscript. We have revised the it according to the referees' comments and suggestions. We want to thank the reviewers for the consutrctive feedback, comments, and suggestions. By addressing their points, we believe that the new version has improved in quality and clarity. The reviewers' original comments, and our responses, are listed below.

Reviewer 1

Dear authors and editors, I am pleased to review this manuscript, which represents a valuable contribution to the growing intersection of deep learning and biological research—particularly from the authors behind the widely recognised DINO framework. The manuscript is well-structured, clearly written, and demonstrates technical competence. Below, I have structured my feedback according to the review format, beginning with a summary of my overall assessment:

This work introduces Cell-DINO, a self-supervised learning approach based on Vision Transformers, adapted from DINOv2 for the extraction of image-based embeddings from cellular microscopy data. The authors claim that these embeddings are "unbiased" and can be applied across a range of biological tasks without the need for manual annotation. The approach is promising and has the potential to significantly advance bioimage analysis and scalable phenotyping.

Key strengths of the paper include:

- The technical contribution of applying and benchmarking DINOv2 in this context. - A thorough empirical evaluation. - Clear writing and a commitment to open science through the public release of code and models.

We thank the reviewer for the constructive feedback and for recognizing that our manuscript is well-structured, clearly written, and demonstrates technical competence.

That said, a few areas require further clarification or improvement:

- The robustness of the embeddings to batch effects remains unconvincing and should be explored in more depth.

We regret the confusion. We do not claim that Cell-DINO is robust to batch effects. In fact, as any other learning method for cellular morphology, it requires batch correction methods to succeed. We have added a new subsection with title "Cell-DINO embeddings require batch correction" to explain this in detail. More discussions below.

- The underperformance of the fine-tuned model (Cell-DINO+) relative to its non-fine-tuned counterpart is unexpected and requires explanation.

We thank the reviewer for pointing this out. We investigated the difference and we found that the fine-tuned models were insufficiently trained with respect to the baselines. We have corrected the experiments and fixed the results in the manuscript (Figure 3). In addition, we report the experimental details in the Methods Section and add more discussions below.

- Benchmarking would benefit from broader comparisons, particularly against recent state-of-the-art SSL approaches (e.g., ref 31).

We appreciate this suggestion. We now have included results with two state-of-the-art models that have been recently released in the field: SubCell (ref 31) and OpenPhenom (ref 36). The results are presented in Section "Cell-DINO outperforms other pre-trained models". SubCell (ref 31) presents orthogonal contributions to our work because their strategy is not purely self-supervised. Specifically it includes a multi-objective loss supervised with cell type, and protein information, combined with a self-supervised reconstruction loss following MAE. The results are reported in Table 2 (more discussions in the main text):

Table 2. Comparison to other state-of-the-art pre-trained models. *Top and middle: F1-scores obtained in the HPA-FoV and HPA-SC datasets, respectively, on the protein localization (PL) classification and cell line (CL) prediction tasks. Bottom: Results using Cell Painting images on the LINCS mechanism-of-action prediction task (MOA AUPRC).*

HPA-FoV	*Pretrain data*	*Architecture*	*PL*	*CL*
OpenPhenom [26]	*RxRx3, JUMP-CP*	*ViT-S*	*23.2*	*81.7*
Cell-DINO	*HPA-FoV*	*ViT-L*	**65.5**	**89.3**

HPA-SC	*Pretrain data*	*Architecture*	*PL*	*CL*
SubCell [18]	*HPA-23*	*ViT-B*	*63.6*	*73.4*
Cell-DINO	*HPA-SC*	*ViT-L*	**78.5**	**86.4**

Cell Painting	*Pretrain data*	*Architecture*	*MOA AUPRC*
OpenPhenom [26]	*RxRx3, JUMP-CP*	*ViT-S*	*6.40*
SubCell [18]	*HPA-23*	*ViT-B*	*7.65*
Cell-DINO	*Comb Cell Painting*	*ViT-S*	**7.67**

- The paper should provide more details regarding computational resource requirements to improve accessibility for a wider range of researchers.

While it is expensive to train a ViT in general, using DINOv2 as the training strategy is theoretically more expensive than other strategies by a factor not greater than two. We added the following clarification to the Methods Section:

The total training cost of a ViT is associated with three main components: 1) image resolution choices, 2) model size, and 3) training dataset size. Image resolution imposes a cost proportional to image and patch size: the larger the image and the smaller the patch, the more tokens need to be processed by self-attention modules, resulting in complexity that grows quadratically with the number of tokens. Model size multiplies this complexity by the number of layers and dimensionality of hidden representations, and dataset size multiplies complexity again by the number of training images. These three aspects are common to training any ViT, regardless of strategy (supervised vs self-supervised). Cell-DINO follows the same strategy as DINOv2, which increases the complexity of training the ViTs by a factor of approximately two: it simultaneously trains a teacher and a student network (it is less than two in practice because gradients are only computed for the student network). This is the only major source of additional computational cost in the Cell-DINO algorithm compared to other strategies such as supervised ViTs and MAEs. The implementation of DINOv2 has been optimized using the PyTorch framework making it easy to use multi-GPU parallelization, which facilitates scaling when the computational resources are available.

- Additionally, I noted one minor figure-caption inconsistency and a small wording issue.

Thank you, we carefully proofread the new version of the manuscript.

Overall, I recommend major revisions to improve the manuscript's robustness and practical relevance. If batch effects cannot be mitigated, the method's value for biological applications is limited.

Our results indicate that batch effects can be mitigated. In the same way as single-cell RNAseq data is useful as a trascriptomics measurement and still needs batch correction for downstream analysis, Cell-DINO embeddings are meaningful image-based features that need correction to reduce unwanted variation. In fact, we find that all image-based methods, including the baselines, require batch correction in order to succeed. We report our findings the new subsection "Cell-DINO embeddings require batch correction".

We quantitatively evaluate the difference between using Cell-DINO embeddings with and without batch correction on the MoA prediction task with the LINCS Cell Painting dataset. In this experiment, single-cell embeddings are aggregated hierarchically using the mean of features per field of view, then per well, and finally per treatment. Sphering [1, 28] is used as the batch correction procedure right after per-well data aggregation, and before treatment-level aggregation. The results indicate that the ability to predict MoAs is reduced by 28% without batch correction (Table 4), resulting in a significant degradation of performance. We used the same procedure to measure the impact of not batch-correcting data in feature embeddings learned by other methods. The results in Table 4 confirm that all image-based embeddings exhibit a similar degradation in performance when batch effects are not addressed (see Supplementary Material). In this case, batch effects are a negative bias, but in other cases, they can be a positive bias that overestimates predictive performance. Either way, the best practice is to always test for the presence and impact of batch effects to prevent confounded results. Batch effect correction for imaging data has witnessed remarkable progress recently [33, 3], but it remains an open problem in the general case, and more research is necessary to design effective and robust batch correction algorithms.

Table 4. *Model Performance Comparison With and Without Batch Correction (B.C.) on the LINCS Cell Painting dataset. The downstream task is MoA classification and the performance metric is PR-AUC. The reported value is the mean of 10 experiments.*

Model	With B.C.	Without B.C.	Percent drop
CP-CNN [28]	7.32 ± 0.21	5.54 ± 0.17	-24.26%
OpenPhenom [26]	6.40 ± 0.15	4.09 ± 0.25	-36.10%
SubCell [18]	7.65 ± 0.17	6.18 ± 0.19	-19.23%
Cell-DINO	7.67 ± 0.29	5.48 ± 0.33	-28.45%

Even if they can, the degradation in performance after fine-tuning suggests that users may need to retrain from scratch, creating a barrier for adoption in resource-constrained labs.

Thank you again for pointing out the degradation of performance with finetuning. It has been addressed as mentioned above, and the results are reported in Figure 3, as follows:

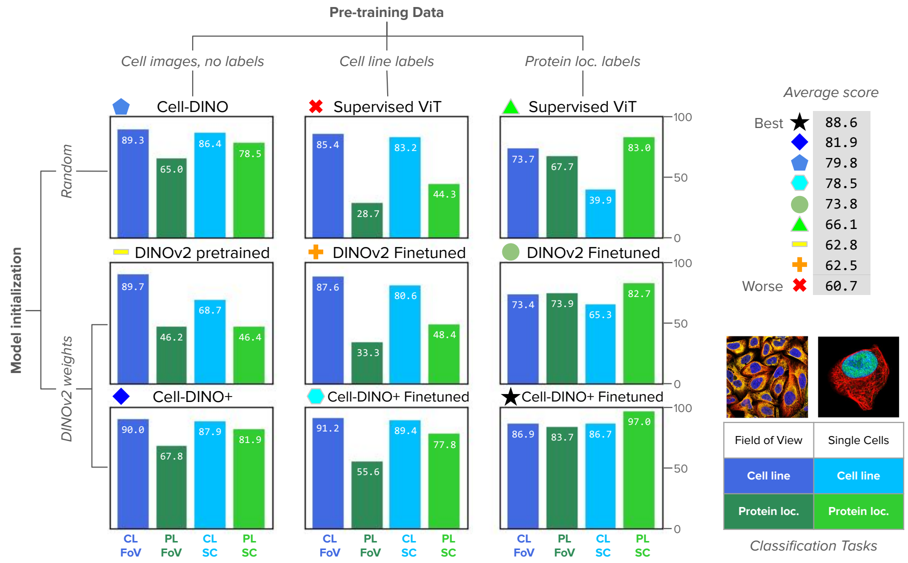

Figure 3. ***Performance evaluation of Cell-DINO on the HPA dataset.*** *All reported results are the F1-score of the corresponding classification task. There are two classification tasks: protein localization (PL / blue shades) and cell-line (CL / green shades) prediction. Each task is evaluated in two versions of the HPA dataset: field-of-view (dark colors) images and single-cell images (light colors). See legend in the bottom-right. Results are presented in a grid of bar-plots colored according to the task and dataset, and organized by pretraining data (columns) and model initialization (rows). Pre-training data goes from no supervision with any labels (left column), to medium supervision with cell line labels (center column), and strong supervision with protein localization labels (right column). The rows consider random initialization (top row), pre-trained DINOv2 models without self-supervision with cell images (center row), and initialization with DINOv2 pre-trained weights with additional self-supervision with cellular images (bottom row). The average score in the top-right corner of the figure identifies each method with a colored icon according to the ranking from best to worse performance. The results of Cell-DINO finetuned are reported in the Supplementary Material.*

In addition, fine-tuning can *always* improve performance on one task, but it requires labels and computing power, as noted by the reviewer. More critically, after fine-tuning, the model loses the ability to perform well in other potential tasks. We conducted fine-tuning experiments to show how the representation may degrade in other tasks when supervision is introduced. We still recommend to use self-supervised Cell-DINO models and instead of fine-tuning them, use linear or nearest-neighbors classifiers to solve diverse tasks with minimal computational cost.

That said, if these issues—particularly batch effects and fine-tuning—are clearly ad-

dressed or thoroughly discussed, the work will still represent a valuable contribution, even without additional experiments.

We thank the reviewer for suggesting a thorough discussion of these issues, which have improved the clarity and scope of our work. Please, see more details below.

- What are the main claims of the paper and how significant are they for the discipline?

The paper introduces and demonstrates Cell-DINO, a self-supervised learning approach based on Vision Transformers and adapted from the DINOv2 algorithm. It is designed to generate rich, ”unbiased” image-based embeddings of cellular morphology from microscopy images, with the goal of enabling scalable and versatile phenotyping without manual annotations. The method is intended to support a wide range of downstream biological analyses, such as protein localization, compound screening, and the recognition and quantification of morphological variations in cells.

These claims have the potential to be highly significant for the field of bioimage analysis, provided that the authors can convincingly demonstrate that these ”unbiased embeddings” are indeed robust to confounding factors such as batch effects, which could otherwise limit the method's validity.

We regret the confusion. In our earlier version, we used the term ”unbiased embeddings” to mean that the embeddings are not targeted to a specific morphological trait or phenotype, but rather, are broadly useful for characterizing many morphological features. We did not intend to claim that the features learned by Cell-DINO are robust to batch effects, or that the term unbiased means without batch effects. To prevent this confusion, we have removed the *unbiased* term from much of our manuscript, starting from the title, and only use it when it is strictly necessary in context. In addition, we now openly discuss batch effects related problems in a new subsection in the manuscript ”Cell-DINO embeddings require batch correction”.

While the authors apply Harmony to account for biological variation (specifically cell-line-dependent variation), this post hoc correction does not fully address broader technical batch effects (e.g., plate-to-plate or lab-to-lab variability). I would encourage the authors to address this aspect explicitly. For instance, prior studies such as ref 29 acknowledge batch effects, ref 36 incorporates mitigation strategies, and ref 46 attempts to analyze technical biases. In large-scale efforts such as the Human Protein Atlas (HPA), where this method could be highly valuable, ensuring robustness to batch effects is essential.

We thank the reviewer for pointing this out. In line with previous works, we now explicitly acknowledge the presence of batch effects in the features learned by Cell-DINO in the new subsection “Cell-DINO embeddings encode technical variation”.
Batch correction is an open problem in imaging, and there is no clear consensus on how to prevent and remove batch effects yet (experimental preventions vs computational correction?). To discuss these issues openly, we now expand the discussions in the Supplementary Material. Specifically, we added a qualitative evaluation for the HPA dataset used in

our study as follows:

> *Cell-DINO embeddings display signs of batch effects in both the HPA and the CPG datasets. This can be observed qualitatively in Figure S1A for the HPA dataset, where some cell-line clusters exhibit fragmented subclusters in relatively distant neighborhoods. For example, the cell lines U251MG, U2OS, and A431 have three major well-aligned clusters, and three equally aligned minor clusters in the center of the plot. The fragmentation could be the result of batch effects, but to verify objectively, batch information is necessary (not fully available for the HPA Kaggle datasets used in this study). Nevertheless, we show how Cell-DINO embeddings can be transformed to reduce unwanted variation by using a popular batch correction algorithm from the single-cell genomics literature [25] to remove one prominent and known source of variation: cell line identity. The result in Figure S1C shows that the transformation successfully integrates the data and reveals other biologically relevant information (protein localization) encoded in the Cell-DINO embeddings. This does not fully address batch effects in this example, but illustrates how Cell-DINO embeddings encode rich phenotypic information that can be recovered with appropriate transformations.*

The manuscript mentions that ”augmentations are used to indicate which information is not important,” but it does not appear that these augmentations were designed specifically to mitigate batch variation.

The reviewer is correct. The augmentations are not designed to remove batch variation. We corrected the statement to indicate that augmentations are designed to introduce valid image alterations and make the features robust to such changes. However, the augmentations are not meant to simulate technical variation or batch effects, because these are hard to replicate computationally. We paraphrased to clarify in the Methods section as follows:

> *The images that the two networks receive are views of the same sample, and augmentations are used to introduce low-level signal alterations that prevent encoding trivial features.*

Additionally, manually selecting augmentations may introduce human bias at a different level, which slightly contrasts with the paper’s emphasis on minimizing bias stemming from human preconceptions. For instance, Figure 2C shows multiple clusters of mitochondria and others, potentially hinting at unexplored batch or technical variation.

The augmentations in Cell-DINO are designed to account for low-level signal variations (intensity, contrast) or trivial transformations (flips, rotations). In that way, there is no human bias related to phenotypic variation or biological guidance of any type. The mitochondria cluster indicated by the reviewer could indeed be a problem related to technical variation. We used the HPA data available through Kaggle, which unfortunately does not have plate and well information to verify this conclusively. However, we add more discussions in the supplementary material, together with a figure that highlights these potential

issues, as follows:

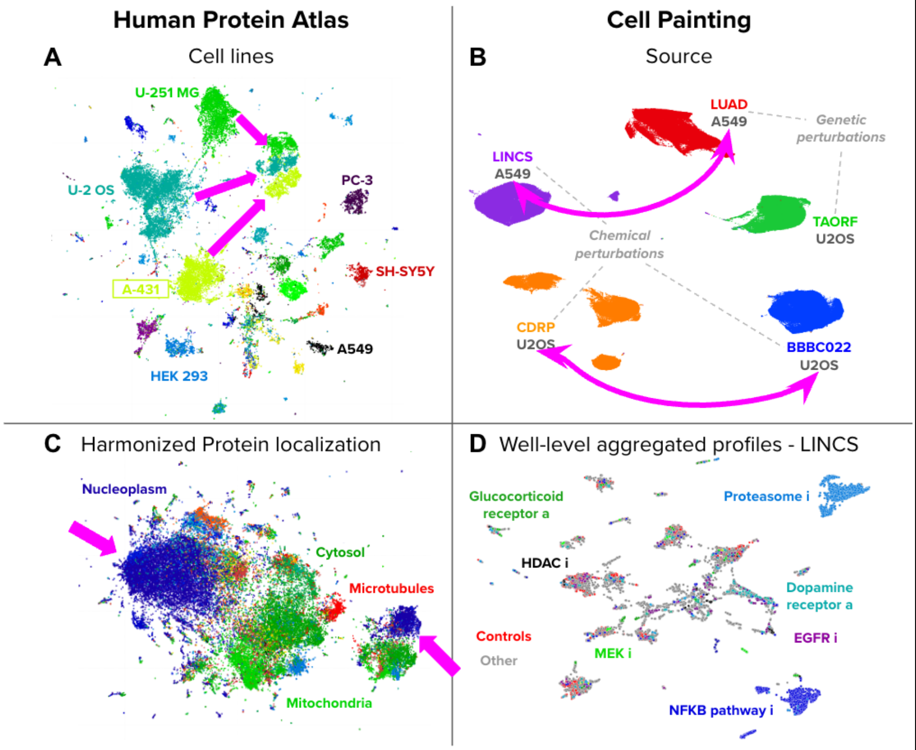

Figure S1. ***UMAP visualizations of image-based embeddings obtained with Cell-DINO.*** *Pink arrows indicate possible clustering due to batch effects. Left column: Image-based embeddings of the HPA-FoV dataset. Right column: Image-based embeddings of the Cell Painting datasets. Top row (A,B): unprocessed embeddings. Bottom row (C,D): transformed embeddings for downstream analysis.*

Furthermore, the broader impact of this work will depend on its accessibility to the biological research community. Its significance hinges on whether pre-trained models can be directly applied by other laboratories or whether retraining on new datasets is feasible with widely available computing resources.

We agree with the reviewer. The pre-trained models are shared and made open to the community to facilitate this exploration. As part of our concurrent work, we have also released other DINO models that are channel-agnostic and may be usable right-away by other labs that cannot train their own. We believe that Cell-DINO can help scientists advance their biological experiments with high-quality embeddings. We added the following recommendations in the Supplementary Material:

Recommendations for adopting Cell-DINO

Cell-DINO requires GPU acceleration, which are becoming more accessible to labs around the world as AI infrastructure keeps growing in academia and government. We encourage scientists to experiment with Cell-DINO in the following way:

1. *First, explore pre-trained Cell-DINO models in the specific application of interest. The models can be used out-of-the-box for feature extraction if the number of channels matches. If not, explore the next options.*
2. *If the number of channels do not match, explore our recently released DINO-BOC model [13], which is channel-agnostic. It has been trained with the same principles of Cell-DINO to process one channel at a time.*
3. *Design a benchmark to measure success. This is highly specific to the application of interest, and may or may not require labels.*
4. *After running the benchmark with Cell-DINO embeddings, compare against simpler approaches as a sanity check. For instance, use CellProfiler features or other models released by the community.*
5. *If the results are not satisfactory, explore training your own Cell-DINO model. Start training a ViT-small model and continue benchmarking to evaluate whether the new results are successful or not. Growing the model size may improve performance, but it also requires more resources or time to converge.*
6. *We recommend initializing weights with DINOv2 or Cell-DINO weights, as our experiments indicate that model initialization can improve performance. However, this is not required, and needs to be guided by the benchmark.*

Cell-DINO can be helpful in many applications, but it also requires resources, time, and expertise. As the community makes progress with training, validating, and releasing more models, its usability and accessibility will increase over time.

At present, there is no mention of the computational resources required. Based on the model complexity, it appears that substantial infrastructure (e.g., approximately 8 A100 GPUs running for two weeks per model) and expertise would be necessary. I recommend that the authors explicitly report training requirements and suggest ways to lower the barrier for adoption.

We now report the details of computational resources used in our experiments in a new section in the Methods Section, with title 'Computational resources used for training Cell-DINO models'. In addition, we have added explanations about the theoretical cost, and recommendations to reuse Cell-DINO models. If re-training is necessary by an objective metric, we recommend training with a ViT-small as a start, which can be computationally less intensive than the models in this work. The added details are as follows:

In our experiments, we used standard image resolution of 256x256 pixels for the HPA datasets, and 128x128 pixels for the CPG datasets. Patch size is 16x16 pixels in both cases. We trained ViT-large models, and the sizes of the training datasets are: HPA-FoV 200K images, HPA-SC 500K images, and combined Cell Painting 5M images. For pretraining, Cell-DINO ran for about 12 hours on 32 V100-32G GPUs on the HPA-FoV dataset, and 21 hours for the HPA-SC dataset. On the combined Cell Painting dataset, Cell-DINO ran for 11 hours on 64 V100-32G GPUs. The cost of using a trained Cell-DINO model is the same as any other ViT as there is no additional pre- or post-processing required. We ran feature extraction on 8 GPUs, which typically takes one hour for 0.5M images. We also experimented with training ViT-small models in the exploration phase, which significantly reduces the computational needs by approximately a factor of four. This means that we can train a Cell-DINO ViT-small model with the combined Cell Painting dataset (5M 128x128 images) in approximately 12 hours using 8 GPUs.

- Are these claims novel? If not, which published articles weaken the claims of originality of this one?

The application of DINOv2 to biomedical imaging data, specifically in cell microscopy, alongside benchmarking, constitutes a technically meaningful and relatively new contribution. I appreciate the authors' effort and investment in adapting a state-of-the-art algorithm like DINOv2 for bioimage analysis and for providing a thorough empirical evaluation.

We thank the reviewer for appreciating the novelty of our work in the field.

That said, the broader claim—that self-supervised learning (SSL) outperforms classical feature engineering and supervised methods for microscopy image analysis—is well-supported in prior literature and is cited. Furthermore, the use of transformer-based SSL in bioimage analysis is not entirely novel, as similar approaches have been explored (e.g., with DINO, SimCLR, and MAE), although DINOv2 itself has not been applied in this domain. In summary, while the technical contribution of applying and benchmarking DINOv2 is valuable, the conceptual advancement is relatively incremental when compared to existing SSL work in bioimage analysis.

We agree with the reviewer's assessment. Specifically, transformer-based SSL has been explored in other works, including a prior version of this manuscript (pre-printed back in 2023). However, there is confusion in the field when the literature calls certain methods self-supervised when they actually use supervision with annotations and prior experimental knowledge. The main claim of our paper is that feature embeddings learned *exclusively* with images in a self-supervised way are not biased by existing pre-conceptions, and can be as good or even better than supervised approaches.
In this study, we aim to provide clarity by systematically benchmarking SSL algorithms and supervised models. We found that Cell-DINO is the best algorithm for *purely self-supervised* learning of cellular features. Other models may offer better performance in

specific tasks, but these have been trained with additional annotations, metadata, or biological information, making them strongly supervised, weakly supervised, or dependent on prior knowledge. We argue that for frontier imaging types and discovery studies where such annotations or knowledge may not be available, Cell-DINO can offer strong features.

If the authors could demonstrate that these embeddings can be directly clustered into biologically meaningful groups without further correction, the novelty would be more pronounced. As it stands, the term ”unbiased” seems to primarily refer to the absence of manual labels, rather than addressing technical confounding factors like batch effects.

In this work, we present a carefully conducted study that documents the unique and emerging properties of DINO models when trained on cellular images. We emphasize that the term unbiased was meant to indicate that the algorithm is 100% self-supervised and does not require any type of biological knowledge or domain guidance to produce useful and meaningful features. This makes our approach different from others, which still need supervision to succeed.
We regret the confusion with batch effects, and we clarify that Cell-DINO features are also prone to batch effects and can benefit from post-hoc correction. In fact, they need post-hoc correction in the same way as scRNAseq or other transcriptomics data also require correction. Being prone to batch effects does not limit the usefulness of these measurements, and it is the same situation with DINO features.

- Are the claims properly placed in the context of the previous literature? Have the authors treated the literature fairly?

The paper references relevant prior work, but the comparative discussion could be expanded. For example, there is no direct comparison between Cell-DINO and the implementation in ref 29, which would be highly relevant. Ref 31 benchmarks against ref 29 as well as against an earlier version of this submission.

The focus of our work is to evaluate how much information we can extract from images without introducing any type of guidance or labels, because in some imaging applications this information may not be available or may not be reliable. Therefore, we present comparisons among Cell-DINO, MAE, and SimCLR in our experiments with HPA (see Fig. 3, and Tables 1,2,3) and Cell Painting data (see Fig. 6 and Tables 1,2,4). We prioritized these baselines because the algorithms are purely self-supervised, and image-based only without requiring additional domain information. From the scientific point of view, this makes an apples-to-apples comparison between methods in terms of access to information and supervision levels. Ref 29 is not purely self-supervised because it leverages domain knowledge (protein identity) to improve performance (see Fig. 4 in their paper, Cytoself results without protein id. pretext task), and it does not have a pre-trained model to compare against.
We include comparisons to methods that have access to more information when their pre-trained models are available. That includes strongly supervised models (bestfitting) and weakly supervised baselines (CP-CNN) for HPA (Figure 4) and for Cell Painting (Figure

6), respectively. In this revision, we now include new comparisons with two other recently released pre-trained models in Table 2, including SubCell (ref 31) and OpenPhenom (ref 36). Note that SubCell is weakly supervised as it leverages information of cells and proteins to learn representations. OpenPhenom, on the other hand, is a channel adaptive MAE without additional domain supervision. The results indicate that Cell-DINO still yields better performance in all tasks.

Table 2. **Comparison to other state-of-the-art pre-trained models.** *Top and middle: F1-scores obtained in the HPA-FoV and HPA-SC datasets, respectively, on the protein localization (PL) classification and cell line (CL) prediction tasks. Bottom: Results using Cell Painting images on the LINCS mechanism-of-action prediction task (MoA AUPRC) [37].*

HPA-FoV	Pretrain data	Architecture	PL	CL
OpenPhenom [26]	RxRx3, JUMP-CP	ViT-S	23.2	81.7
Cell-DINO	HPA-FoV	ViT-L	**65.5**	**89.3**
**HPA-SC**	**Pretrain data**	**Architecture**	**PL**	**CL**
SubCell [18]	HPAv23	ViT-B	63.6	73.4
Cell-DINO	HPA-SC	ViT-L	**78.5**	**86.4**
**Cell Painting**	**Pretrain data**	**Architecture**	**MoA AUPRC**	
OpenPhenom [26]	RxRx3, JUMP-CP	ViT-S	6.40	
SubCell [18]	HPAv23	ViT-B	7.65	
Cell-DINO	Comb Cell Painting	ViT-S	**7.67**	

In addition, we expanded the evaluation with Cell Painting data to include new results with SubCell and OpenPhenom in Figure 6 as follows:

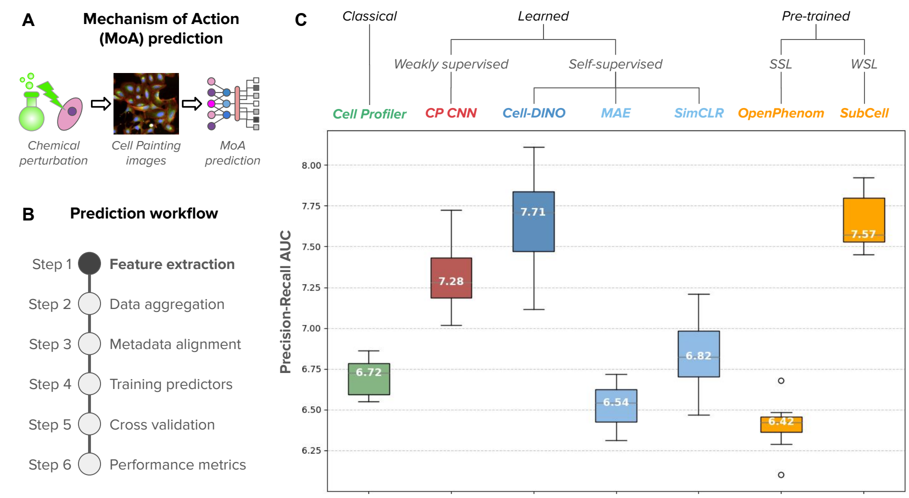

Performance evaluation of Cell-DINO on the CPG datasets. *A) The evaluation task is mechanism of action (MoA) prediction, where chemical compounds are used to treat cells and Cell Painting images are collected to assess their effect and infer their MoA with computational models. B) Illustration of the steps of the computational workflow to predict MoA from Cell Painting images. The only step that changes in these experiments is feature extraction (highlighted in bold). C) Box plots comparing the distributions of performance for the evaluated feature extraction methods. Horizontal axis: evaluated methods organized by feature type. Vertical axis: performance score according to the area under the precision-recall curve of the multi-class MoA prediction problem [37]. Highlighted numbers in the boxes are the median values of the distribution.*

Additionally, ref 46 trains DINO, MAE, and SimCLR on Cell Painting data, but the authors do not compare Cell-DINO to those results.

The reviewer is correct that ref 46 trains DINO, MAE, and SimCLR on Cell Painting data, in the same way as we do. We consider their results consistent with our experiments, as they also independently confirm that DINO yields better results than competing self-supervised strategies. The main difference between their results and ours is the specific Cell Painting subset used (ours is LINCS Cell Painting and theirs is JUMP Cell Painting), but other than that, there is no difference in methods between their approach and ours. We leave evaluations of Cell-DINO on the JUMP Cell Painting dataset for future work, as the scale of the dataset and computational resources required to run these experiments can be significant and beyond the purpose of this study.

In particular, Cell-DINO could be a powerful tool in the context of industrial-scale Cell Painting datasets such as the JUMP-consortium and RxRx-series of datasets [9, 15], which we leave as a future research direction.

It is also noteworthy that ref 31 made concerted efforts to outperform the earlier Cell-DINO submission. It would strengthen the manuscript if the authors discussed how Cell-DINO compares not only to their own baselines (including an MAE trained with augmentations optimized for Cell-DINO) but also to these newer, closely related methods, including the MAE from ref 31.

We appreciate that the SubCell team has found our previous preprint useful for their evaluation. We have included a comparison of their model in our evaluation and have found that the SubCell model yields strong performance and is highly competitive to Cell-DINO. We note that SubCell leverages domain knowledge during training, while ours is 100% image-based only. We have added comparisons to SubCell in Table 2 and in the Supplementary Material. The fact that DINO is competitive with specialized models like SubCell is a strength of our approach. We added to the discussions the possibility of combining such domain knowledge to train Cell-DINO models in a multi-task setting, potentially improving performance as well. The following are the results reported in the Supplementary Material:

Table S1. *Cell-DINO compared with Subcell's baselines on the protein localization task of HPAv23 with the 19 classes used in the Kaggle competition.*

Method	*Single Cell (MAP)*	*FOV (MAP)*
MAE-CellS-ProtS-Pool	*60.0*	*67.4*
ViT-ProtS-Pool	*61.3*	*70.0*
Cell-DINO	**66.4**	**71.4**

Table S2. *Cell-DINO compared with Subcell's baselines on the protein localization task of HPAv23 with 31 classes.*

Method	*Single Cell (MAP)*	*FOV (MAP)*
MAE-CellS-ProtS-Pool	*39.9*	*46.0*
ViT-ProtS-Pool	*42.1*	*51.2*
Cell-DINO	**44.2**	**51.4**

More details about these experimental results are reported in the Supplementary Material.

- Do the data and analyses fully support the claims? If not, what other evidence is required?

The current data and analyses support several claims regarding model performance. However, there is no direct evidence that the embeddings are robust to batch effects or other technical biases. As mentioned earlier, prior works (e.g., ref 29, ref 36, ref 46) provide examples of how to analyze and mitigate such issues.

We clarify that the Cell-DINO features are not expected to be robust to batch effects.

Our experiments also include examples of how to mitigate that variation, and we included a new section in the main text to report the quantitative results in the subsection "Cell-DINO embeddings encode technical variation". We also have added qualitative results in the Supplementary Material.

In addition to clarifying the terminology (e.g., "unbiased"), I would encourage the authors to explore whether augmentations or alternative strategies could reduce batch effects. Such an investigation could represent a meaningful contribution to the field. If no such strategies are identified, this may suggest that DINO, in its current form, may not be fully optimal for tasks requiring invariance to batch variation.

We agree with the reviewer that the term "unbiased" was confusing and we have removed it from the manuscript. Also, we appreciate the reviewer's suggestion to explore if batch effects can be mitigated with image augmentations. We conducted exploratory experiments in that direction and found no evidence that data augmentation helps in reducing batch effects. After careful investigation in the literature, we also found studies reporting that domain-specific augmentations are not needed in self-supervised learning [29]. We added clarifications to Methods section as follows:

> *Note that augmentations in our work are not meant to address batch effects. In our experiments, we explored the role of basic augmentations as a means to improve performance, but not necessarily remove unwanted technical variation. We did not find evidence that these augmentations make the models robust to batch effects. Prior work has shown that domain-specific augmentations may not be as critical as previously thought for self-supervised learning [29]. In addition, generative models and style transfer techniques have been explored to produce augmented data that explicitly target batch effects [33], which is a more promising approach to mitigate technical variation. We did not explore this approach in our study, and we leave this possibility for future work.*

- Would additional work improve the paper? How much better would the paper be if this work were performed and how difficult would it be to do this work?

Yes, additional work would enhance the paper, both in terms of technical rigor and its practical utility for the broader research community.

1. Computational cost and accessibility (low effort): The authors should explicitly report the computational resources required to train, fine-tune, and deploy Cell-DINO. It would also be helpful to clarify whether these demands are comparable to DINOv2 and to discuss whether the method is primarily suited to well-resourced laboratories or can be more broadly adopted through pre-trained models. Additionally, in Figure 4, while comparing against a single CNN from a top ensemble is reasonable, a direct comparison with the full ensemble in terms of computational cost would provide a fuller picture of performance vs. resource trade-offs.

We have added the computational resources used in our experiments in the Methods

Section, "Computational resources used for training Cell-DINO models". We also clarify that the computational demands are the same as DINOv2, and explain the additional cost of the algorithm compared to other strategies in the Methods Section "Computational complexity".

Regarding the adoption by the community, the DINOv2 algorithm remains accessible to the wider community by virtue of its open source, optimized code base, as well as its low computational complexity and moderate resource needs. This is exemplified by the community itself, who has trained DINO and DINOv2 models on their own for many different types of images, including bioimaging. This is in contrast to other areas of research, such as large language models, where accessibility remains exclusive to very large industry labs only.

Finally, comparing against the full ensemble does not apply to our evaluation, because our work is focused on the analysis of features rather than classifier outputs. Features from an ensemble cannot be aggregated as their embeddings are trained independently and are not compatible even if they have the same dimensionality. There is no doubt that the full specialized ensemble of classifiers is more accurate, and we do not claim to be more efficient or more accurate than their approach.

2. Clarification of fine-tuning effects (low effort): The manuscript should explain why the fine-tuned Cell-DINO+ model underperforms relative to the non-finetuned version (as shown in Figure 3). This raises important questions about whether fine-tuning may, in some cases, degrade learned representations, and whether users would be better served by using the released model as-is or by retraining from scratch.

We agree with the reviewer that this result is unexpected. After carefully investigating the experimental settings, we found that the lower performance of Cell-DINO+ on the Cell Line (CL) classification task in the HPA single-cell (SC) dataset was due to an insufficient number of iterations in the fine-tuning process. The dataset contains about 500K samples, and the non-fine-tuned models are "seeing" about 2M samples during pretraining. However, for these fine-tuning experiments, we had to lower the batch size, resulting in only 800K samples total because we did not adjust the iterations accordingly. We have corrected this issue by training both approaches for the same number of iterations (2M). We also double-checked that the other baselines were trained using a sufficient number of iterations: 2M for the DINOv2 finetuned baseline, and 3.5M for the supervised ViT results (a lower number of iteration was leading to worse results). The results have changed as follows:

Table. F1-scores of Cell-DINO+ model: new result vs previously reported result.

Finetuning labels	*Task*	*Old result*	*New Result*	*Non-finetuned*
Cell-line	*Cell Line*	*84.0*	*89.4*	*87.9*
Cell-line	*Protein Loc.*	*79.2*	*77.8*	*81.9*
Protein localization	*Cell Line*	*87.3*	*86.7*	*87.9*
Protein localization	*Protein Loc.*	*91.1*	*97.0*	*81.9*

We now report the corrected results in the manuscript (Figure 3), and all other details in the Methods Section.

3. Benchmarking and baseline selection (moderate effort): The benchmarking could be strengthened by including direct comparisons to more recent or competitive methods, such as those presented in ref 31, which outperforms an earlier version of Cell-DINO. Additionally, clarifying the rationale for selecting MAE and SimCLR as baselines would be helpful. Addressing the omission of other SSL methods, such as MoCo, BYOL, or SimSiam, would provide reassurance that the chosen baselines are representative of the broader SSL landscape.

We have included direct comparisons with the SubCell and OpenPhenom models in the new version of the manuscript. The focus of our work is image-based self-supervised learning, so we mainly consider baseline algorithms that use only images for learning. In addition, we added our rationale for why the baseline models were selected for evaluation in the main text, as follows:

> *We compared Cell-DINO to other two main families of SSL methods, including reconstructive autoencoders, and contrastive learning. The Mask Autoencoder (MAE) [20] approach uses an image reconstruction objective and has been previously shown to obtain scalable results on cellular imaging tasks [26]. The SimCLR approach [10] introduces a contrastive loss maximizing the agreement between two different transformations of the same images in the feature space. MAE and SimCLR are considered strong self-supervised approaches on standard computer vision benchmarks; MAE is widely adopted by the community, and often requires fine-tuning or additional supervision, while SimCLR has been shown to scale well with more data and larger models [11]. Other methods such as MoCo [21] (contrastive), BYOL [17] (self-distilation), and SimSiam [12] (contrastive) fall into one of the main SSL families evaluated in our work, and have been found to perform on par to each other [12], or to be outperformed by DINO [8] on natural image benchmarks.*

4. Batch effect analysis (substantial effort, uncertain success): A deeper evaluation of whether Cell-DINO embeddings encode batch effects would significantly improve the manuscript's biological relevance. Although Harmony is applied to address biological variability, further work is needed to assess and mitigate technical confounders such as batch, plate, or site effects, which are common in large-scale microscopy datasets.

We confirm that batch effects are encoded in Cell-DINO embeddings. In our analysis, we show that batch correction methods can be used to address unwanted variation, both in HPA and Cell Painting experiments. For large-scale microscopy datasets, such as Cell Painting studies, we show that all methods are prone to batch effects, and all methods improve performance after batch correction, including our Cell-DINO embeddings. These results are now reported in the main text in the subsection "Cell-DINO embeddings encode technical variation", and other qualitative analysis is presented in the Supplementary

Material.

5. Augmentation strategy and impact on robustness (moderate-to-high effort, promising impact): Given the importance of augmentations in SSL frameworks like DINO and SimCLR, the authors should justify their choice of using the same augmentation pipeline across all methods and explain how the selected augmentations balance biological variability and technical bias. Investigating whether augmentations (or learnable augmentation strategies) could mitigate batch effects would be a valuable addition.

We now clarify that our choice of data augmentations for cellular images is meant to ensure that the DINO algorithm receives useful image signals, which are not destroyed by aggressive or incompatible data augmentations. Beyond this basic adjustment, we cannot claim that the augmentations used in this work are meant to mitigate technical variation or remove batch effects.

Differences in channel number and image content with respect to natural images require adjustment of augmentations for biological images. This is done primarily to make sure DINO receives useful image signals to learn relevant features for this type of data.

In addition, we run exploratory experiments in the CPG datasets, and we did not find evidence that augmentations make models more robust to batch effects specifically. There are two aspects to consider regarding data augmentations to address batch effects: 1) technical variation is not a trivial source of noise, and cannot be easily simulated with standard data augmentation techniques. Instead, technical variation may need more complex simulation strategies to be addressed, such as the use of generative models and style transfer techniques, which have been explored in the context of cellular imaging before [33]. 2) The need for augmentations is reduced with more data because real samples exhibit realistic technical variation that is more useful for learning than artificial transformations. The selection of data with diverse technical variation was found to be useful in prior work too [28], and we adopted the dataset proposed in that study in our experiments. We now clarify these aspects related to data augmentation in the Methods Section (see comments above).

- PLOS Computational Biology encourages authors to publish detailed protocols and algorithms as supporting information online. Do any particular methods used in the manuscript warrant such treatment? If a protocol is already provided, for example for a randomized controlled trial, are there any important deviations from it? If so, have the authors explained adequately why the deviations occurred?

The training and evaluation protocols are well-documented, and the code will be made available. However, the manuscript would benefit from including detailed documentation on resource requirements and practical guidelines for reproducibility, particularly for laboratories without access to extensive computational infrastructure.

We thank the reviewer for appreciating our effort to make code and protocols accessi-

ble to other scientists.

- Has the author-generated code that underpins the findings been made publicly available? The authors state that the code will be made available on GitHub upon publication.
- Are details of the methodology sufficient to allow the experiments to be reproduced? Yes.
- Is any software created by the authors freely available? Yes.
- Is the manuscript well organized and written clearly enough to be accessible to non-specialists?

Overall, yes. The manuscript is well-structured and clearly written. However:

1. The term ”unbiased embeddings” could be potentially misleading for non-specialists and would benefit from clarification.

We removed the term unbiased from the main text, and now clarify what the meaning is in the context of this study. We specifically highlight that unbiased does not mean robust to batch effects.

2. The word ”adaptation” may not be entirely precise here, as the modifications to DINOv2 appear relatively minor, and this does not fully align with the concept of domain adaptation. The term ”application” might more accurately describe the use of DINOv2 in this context. In interdisciplinary research, ensuring clarity in technical terminology is particularly important to avoid potential misunderstandings among readers from diverse backgrounds.

We thank the reviewer for pointing this out. We replaced the term adaptation for application.

Minor issues: 1. In Figure 4, the caption states ”horizontal lines,” but the lines are vertical. 2. In ”Data Availability”, the phrase ”as the Cell-DINO model weights...” should likely be ”as well as the Cell-DINO model weights.”

We thank the reviewer for these suggestions. We have made the changes accordingly.

Reviewer 2

In their manuscript Moutakani et al. investigate the potential application and advantages of using the DINOv2 model to study cellular morphology. Cellular morphology in bioimages remains a challenging topic and, despite important improvements since the integration of DL in analyses pipeline in recent years, can still greatly benefit from new and advanced methods to improve further. This is attested by the amount of recently published literature and the regular organization of competitions. It also feels important to note that progress made by research teams dedicated to image analysis is sometimes having a hard time reaching biologist research teams due to both usage complexity and difficulty of retraining the models for their own images. The Cell-DINO method presented here provides an interesting solution to bypass the difficulty of producing ground truth datasets. This has the potential of simplifying the application of such a powerful DL method to end users in biology labs. The manuscript compares the Cell-DINO efficiency to 2 other supervised and self-supervised when applied to 2 publicly available image datasets. These results are showing better results, especially in the low training data range which again has the potential of simplifying the uptake of this method by end users. Overall, this manuscript demonstrates that CellDINO is a useful and promising tool for studying biological questions in the context of imaging and the authors are sharing the code in a FAIR format to reproduce their results. However, making CellDINO more readily applicable to other datasets and more easy to reuse for less computer savvy labs could help make it more impactful. Finally, I would consider important to see more models compared, in order to confirm that CellDINO is consistently performing better than supervised and self-supervised approaches.

We thank the reviewer for their thoughtful comments and suggestions. We appreciate that they find our work interesting and with potential, and that the provided suggestions aim to improve clarity and reach for the biological community.

Main comments:
- The code is conveniently shared in a git repository that will be open after publication. It also describes expected run times for a specific hardware for the cell-dino-hpa. This part is clearer and more developed than the cell-painting-eval that would benefit from reaching the same quality level (with direct links to the notebooks, and expected runtimes and hardware).

We thank the reviewer for pointing this out. We have expanded the documentation of the Cell Painting analysis code, which now describes expected run times, hardware used, and clearer pointers to reproducing the results. The following is part of the clarification text added to the README file:

This repository provides a step-by-step guide to successfully run the LINCS Cell Painting evaluation using Cell-DINO. The LINCS Cell Painting evaluation code was originally developed by Way et al. (2022) in their morphology and transcriptional profiling study. The code in this evaluation is adapted from their implementation (available in GitHub) and focused only on the morphological profiling analysis.

Training Cell-DINO models and feature extraction. *This repository does not contain the code to train Cell-DINO models and does not contain code to extract the corresponding feature embeddings from images. Training and feature extraction need to be performed with the DINOv2 implementation provided here (link to be added soon). Please, follow the instructions there to obtain a pre-trained Cell-DINO model for Cell Painting images, or to train your own Cell-DINO model.*
The Cell Painting datasets used in this study are available in the Cell Painting Gallery. To train the Cell-DINO model we employed the Combined Cell Painting dataset (accession number cpg0019-moshkov-deepprofiler) used in the DeepProfiler study. The dataset has 482GB of imaging data and about 8M single cells. Training with this dataset with 8 GPUs takes about 24 hours with a batch size of 256 and 1 million images per epoch for 100 epochs. For the evaluation part, images from the LINCS Cell Painting dataset were used (accession number cpg0004-lincs), which is a compound screen of approx. 1,500 FDA approved drugs. The full image collection is 61TB of imaging data, and we only used one dose at a time for analysis (out of six available doses). Feature extraction at the single-cell level with the Cell-DINO model on the LINCS dataset takes about 2 hours on 8 GPUs.

Expected runtimes are especially interesting since the authors recommends using the CPU version of the TF package to prevent issues with GPU drivers because TF 2.12 is an old version.

The reviewer is correct that the LINCS Cell Painting evaluation uses an old version of TensorFlow (2.12). This code was originally developed by other scientists in a different study and we adopted their implementation to evaluate a real-world downstream task with the Cell Painting dataset. We consider their implementation as an example task that biologists may be interested in approaching with imaging. The code was adapted to facilitate comparisons between models as reported in the main manuscript. We clarify the scope of the workflow and its expected running time in the documentation, as follows:

Evaluation task. *The main focus of this repository is to predict the mechanism of action of compounds and evaluate the ability of feature extraction methods to do this accurately. This repository assumes that features have been already computed, and provides the necessary code to aggregate, batch-correct, train classifiers, and evaluate performance. The whole procedure is a multi-step process which has been automated to be run in the command line without manual intervention. Running the evaluation from scratch can take about 2 to 4 hours using CPU compute only.*

- The code is well presented and organized but do not possess a jupyter notebook implementation or a graphical user interface such as a napari plugin or the one provided by the Cellpose authors. In it's current shape, the reach of this study could remain limited to only labs with highly skilled computer scientists. Would it be possible to try and offer a more graphical approach to limit this technical step as much a possible and maximize the reach of this algorithm?

Our implementation is meant to facilitate large-scale studies, with hundreds of thousands to millions of cells (e.g., HPA with 1M single cells or Cell Painting with 22M single cells). This is usually better approached with batch jobs that run in dedicated servers using the command line. The analyses presented in our work require data science skills, and it is not meant to be a user friendly application yet.

The scope of our work is limited to demonstrating that the Cell-DINO approach provides better morphological features. But to reach the level of performance reported in the paper in a new image-based experiment, models need to be trained, which requires computational resources and data science or machine learning expertise to configure, optimize, and validate the results.

We still believe that many scientists around the world can train their own models and leverage our proposed Cell-DINO approach to advance large-scale imaging studies. Thus, we aim to disseminate these ideas in a timely manner to facilitate progress in the field. However, there is still work to be done to create general purpose Cell-DINO models that are user-friendly and that can be used with any microscopy images. We clarify this in the main text and leave these research directions for our future work:

- Adding more models for comparison and explaining better why their were chosen would reinforce the paper's results.

We thank the reviewer for this suggestion. We have added comparisons with other models, specifically SubCell (ref 31) and OpenPhenom (ref 36) in Table 2, and additional evaluations to SubCell in the supplementary material. The results are as follows:

Table 2. Comparison to other state-of-the-art pre-trained models. *Top and middle: F1-scores obtained in the HPA-FoV and HPA-SC datasets, respectively, on the protein localization (PL) classification and cell line (CL) prediction tasks. Bottom: Results using Cell Painting images on the LINCS mechanism-of-action prediction task (MOA AUPRC).*

HPA-FoV	Pretrain data	Architecture	PL	CL
OpenPhenom [26]	RxRx3, JUMP-CP	ViT-S	23.2	81.7
Cell-DINO	HPA-FoV	ViT-L	**65.5**	**89.3**

HPA-SC	Pretrain data	Architecture	PL	CL
SubCell [18]	HPA-23	ViT-B	63.6	73.4
Cell-DINO	HPA-SC	ViT-L	**78.5**	**86.4**

Cell Painting	Pretrain data	Architecture	MOA AUPRC
OpenPhenom [26]	RxRx3, JUMP-CP	ViT-S	6.40
SubCell [18]	HPA-23	ViT-B	7.65
Cell-DINO	Comb Cell Painting	ViT-S	**7.67**

Also, we expanded the evaluation with Cell Painting data to include new results with SubCell and OpenPhenom in Figure 6 as follows:

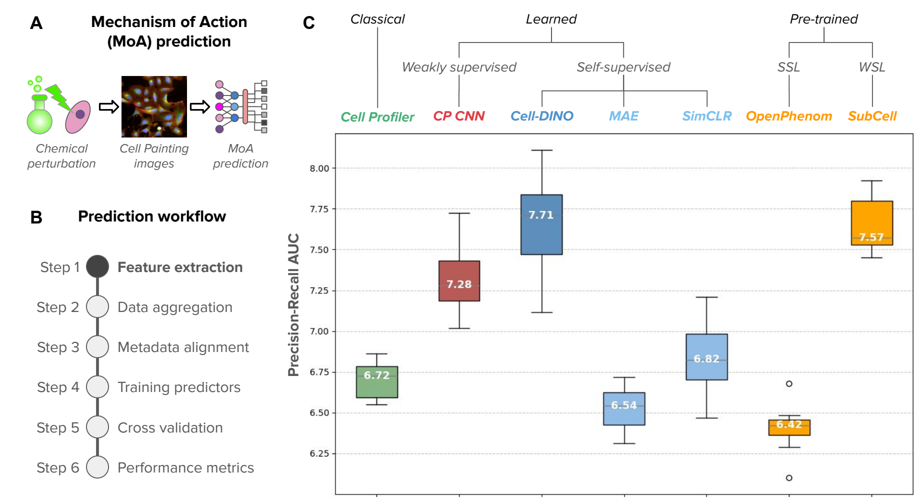

Performance evaluation of Cell-DINO on the CPG datasets. *A) The evaluation task is mechanism of action (MoA) prediction, where chemical compounds are used to treat cells and Cell Painting images are collected to assess their effect and infer their MoA with computational models. B) Illustration of the steps of the computational workflow to predict MoA from Cell Painting images. The only step that changes in these experiments is feature extraction (highlighted in bold). C) Box plots comparing the distributions of performance for the evaluated feature extraction methods. Horizontal axis: evaluated methods organized by feature type. Vertical axis: performance score according to the area under the precision-recall curve of the multi-class MoA prediction problem [37]. Highlighted numbers in the boxes are the median values of the distribution.*

In addition, our evaluation includes comparisons with two types of SSL models, supervised models, and weakly supervised models. While there are many alternatives for learning visual representations in the computer vision and bioimage analysis fields, we try to cover the most representative, as explained now in the main text:

We compared Cell-DINO to other two main families of SSL methods, including reconstructive autoencoders, and contrastive learning. The Mask Autoencoder (MAE) [20] approach uses an image reconstruction objective and has been previously shown to obtain scalable results on cellular imaging tasks [26]. The SimCLR approach [10] introduces a contrastive loss maximizing the agreement between two different transformations of the same images in the feature space. MAE and SimCLR are considered strong self-supervised approaches on standard computer vision benchmarks; MAE is widely adopted by the community, and often requires fine-tuning or additional supervision, while SimCLR has been shown to scale well with more data and larger models [11]. Other methods such as MoCo [21] (contrastive), BYOL [17] (self-distilation), and SimSiam [12] (contrastive) fall into one of the main SSL families evaluated in our work, and have been found to perform on par to each other [12], or to be outperformed by DINO [8] on natural image benchmarks.

Authors summary and introduction

- the authors categorize the "current approaches to analyze cell microscopy images as relying on classical features carefully chosen [...] or supervised strategies". I believe this may have to be updated with the recent [SAM in bioimage analysis paper](https://www.nature.com/articles/s41592-024-02580-4) as well as the (https://www.biorxiv.org/content/10.1101/2024.04.06.587952v1) [Transformers do not outperform Cellpose] paper.

We thank the reviewer for this suggestion. The text has been updated to reflect these changes and progress in the field:

Cellular imaging is widely used in biological studies, and often enables quantitative analysis of phenotypic variation through the use of machine learning algorithms.

In addition, we added the references suggested by the reviewer in the introduction:

Indeed, deep learning now powers robust cell segmentation methods [7, 22, 35, 16, 24, 2, 36], as well as cell phenotyping using classification networks [32, 27].

Cell-DINO encodes diverse biological properties of cells

- In Fig 2B: The subtitle "single cells - training datasets" is unclear. I guess the authors are not talking about the dataset used for training, so what is it meant to describe? In general the figure 2 legend could be more clear. The figure 2D is not used in the main text.

We regret the confusion. We changed the title of Fig. 2B from "single cells - training datasets" to "Colors: studies". Also, we expanded the legend of Figure 2 to include all other missing details, as follows:

A) Points are fields of view, and the colors reveal the cell line of the sample. B) Points are single cells, and the colors reveal the source study where the cells come from: LINCS [38] and LUAD [6] are A549 cells, while CDRP [5], TAORF [34], and BBBC022 [19] are U2OS cells. Samples from the five studies were used for training. C) Points are embeddings of fields of view after harmonization [25]; colors are protein localization labels. D) Points are well-level aggregated features after batch correction with sphering [1] from the LINCS dataset. Colors are mechanisms of action; names with ***a*** *are antagonists and names with* ***i*** *are inhibitors).*

- The claim that "protein localization patterns are largely invariant across human cell lines" should be accompanied by a citation.

We agree with the reviewer. We added the following citation: *Orre, Lukas Minus, et al. "SubCellBarCode: proteome-wide mapping of protein localization and relocalization." Molecular cell 73.1 (2019): 166-182.* In their study, the authors found that approximately 80% of more than 12,000 proteins match localization across 5 cell lines using mass spectrometry analysis. We adjusted the language as follows:

... supporting the idea that protein localization patterns may be consistent across human cell lines [31] (Figure 2C).

- The note that "harmonized HPA-FoV embeddings qualitatively resemble the structures identified by supervised methods" is a very interesting claim, but the authors should make this comparison simpler for the reader by putting both figures side-by-side or, if possible, quantitatively assess it.

We have added a side-by-side comparison of the UMAP visualizations to the Supplementary material, as follows:

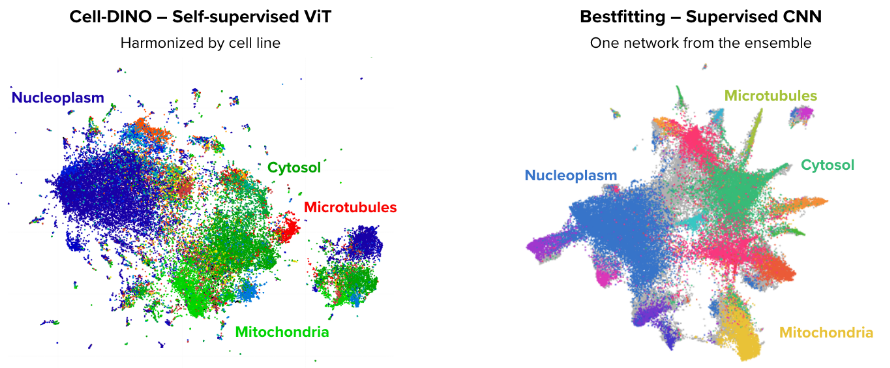

Figure S2. ***UMAP visualizations of image-based embeddings obtained on the HPA-FoV dataset.*** *The self-supervised approach Cell-DINO (left) finds clusters that resemble the clusters learned with supervised learning (right). Four major clusters are highlighted in each map: nucleoplasm, cytosol, microtubules, and mitochondria. The supervised approach was originally reported in [32] and the visualization is available in the Human Protein Atlas website through the following link:* `https://www.proteinatlas.org/humanproteome/subcellular/location+umap` *(accessed August 2025).*

Cell-DINO outperforms supervised ViT results

- The chosen supervised model doesn't seem to be described here. Please describe it in details and explain this choice. In case it is a single model, considering how many such models exist, I'm not sure this comparison is sufficient to support the claim that Cell-DINO generally outperforms supervised ViT.

The reviewer is correct that we only evaluated the standard supervised ViT, leaving out other potential tricks or supervised optimizations that may improve performance. In our work, we follow the implementation of the supervised ViT originally presented in the paper by Dosovitskiy et al. "An image is worth 16x16 words: Transformers for image recognition at scale." ICLR (2021) [14]. We have added the details of our implementation in the methods section as follows:

The implementation of the supervised ViTs used as baselines for the HPA benchmark follows a standard classification strategy where all samples are expected to label annotations and a loss function minimizes the classification error [14]. Loss function: The training objective minimizes the softmax cross-entropy loss. Optimizer: The supervised ViTs were trained using the AdamW optimizer with parameters $\beta_1 = 0.9$ *and* $\beta_2 = 0.999$*. Learning rate schedule: The learning rate was gradually increased over the first few epochs (warmup) and then linearly decayed for the remainder of training. Data Augmentation: the same augmentations used for self-supervision were employed here.*

In addition, we clarify in the main text as follows:

we find that Cell-DINO models can outperform standard supervised ViT models by a substantial margin. Our supervised implementation follows the original classification strategy for ViTs [14], leaving out other potential tricks or advanced supervised optimizations that may improve performance.

- Considering that Cell-DINO+ outperforms Cell-DINO in both pre-trained and fine-tuned conditions for protein-localization, could the authors discuss why they favor Cell-DINO with random initialization?

We report that Cell-DINO+ performs better than Cell-DINO as an interesting observation. But we report other experiments using Cell-DINO with random initialization to keep a fair comparison with other models. In practice, a Cell-DINO+ model is trained with more data because the DINOv2 pre-training had access to more than 150M training images, while the other models only use the available cellular imaging in this study. To isolate the real causes of improved performance, we keep experiments as clean and as comparable as possible to support our claims. We add the following clarification to the main text:

This result points to an easy way of improving self-supervised results, and we highly recommend its adoption in practice. In the rest of this paper, we compare other baselines and strategies against Cell-DINO instead of Cell-DINO+ because the latter has the unfair advantage of being pre-trained with more data (150M+ natural images) which other methods do not have access to.

- The figure lacks a way to compare Cell-DINO results fine-tuned with Cell lines or Protein loc. to Cell-DINO+ to fully be able to understand the effect of the model initialization

We only included Cell-DINO+ finetuned results in the figure to inform on the best performance that could be achieved by combining DINOv2 pretraining and finetuning, and to facilitate the presentation and readibility of the results. For completeness, we also fine-tuned Cell-DINO models, and as expected, the results show that fine-tuning improves performance compared to basic Cell-DINO , while remaining slightly worse than Cell-DINO+ fine-tuned:

Task	*Cell-DINO*	*Cell-DINO ft*	*Cell-DINO+*	*Cell-DINO+ ft*
Cell line classification	*83.3*	*90.6*	*90.0*	*91.2*
Protein localization	*65.0*	*81.9*	*67.8*	*83.7*

We now include these results in the supplementary material and refer to them in the main text.

Cell-DINO is competitive against highly tuned models

- The BestFitting and DualHead solutions described in the text are not highlighted in the figure. Instead ”Kaggle” is displayed as ”a single supervised CNN”. Please make these solutions clearly appear.

Thanks for pointing this out. We change the “Kaggle” term for “BestFitting” and ”DualHead” in the corresponding labels of the figure, which are the models that we use in this evaluation.

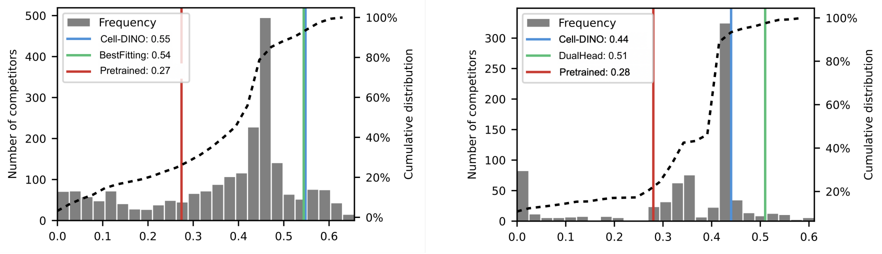

Cell-DINO outperforms alternative self-supervised strategies

- Considering that Cell-DINO heavily outperforms MAE but not so much simCLR, it would be interesting to have more models to compare here.

When it comes to purely self-supervised strategies, there are a few fundamental principles and many algorithms that belong to the same family. We do not consider methods that use additional supervision as our goal is to determine the extent to which we can extract information without annotations. We have expanded the rationale for why the chosen baselines in our evaluation were selected, and why we believe they cover representative strategies for purely self-supervised learning in the computer vision literature. This was added to the document as follows:

We compared Cell-DINO to other two main families of SSL methods, including reconstructive autoencoders, and contrastive learning. The Mask Autoencoder (MAE) [20] approach uses an image reconstruction objective and has been previously shown to obtain scalable results on cellular imaging tasks [26]. The SimCLR approach [10] introduces a contrastive loss maximizing the agreement between two different transformations of the same images in the feature space. MAE and SimCLR are considered strong self-supervised approaches on standard computer vision benchmarks; MAE is widely adopted by the community, and often requires fine-tuning or additional supervision, while SimCLR has been shown to scale well with more data and larger models [11]. Other methods such as MoCo [21] (contrastive), BYOL [17] (self-distilation), and SimSiam [12] (contrastive) fall into one of the main SSL families evaluated in our work, and have been found to perform on par to each other [12], or to be outperformed by DINO [8] on natural image benchmarks.

In addition, we compare to other recently developed methods specialized for cellular image analysis, and report the results in detail in a new section: ”Cell-DINO outpuerforms other pre-trained models” (see below).

- When claiming that Cell-DINO improve upon the performance of state-of-the-art approaches, please cite the results directly in the manuscript

We have added results with two additional state-of-the-art models to compare performance in the tasks evaluated in our work: SubCell (ref 31) and OpenPhenom (ref 36). The new results are reported in Table 2 and Figure 6 with discussions added in the new section “Cell-DINO outperforms other pre-trained models”, as mentioned before.

Cell-DINO reduces the dependency on manual annotations
- In the HPA SC context: the conclusions of this paragraph are confusing, although I agree that the Fig.5 supports the claim of better results in the low training data range, it’s unclear where the 24% and 72% relative improvements are calculated from. It seems to me that the results are fetched from both the protein and cell line graphs and always picking the most striking difference only, in that case it may seem unfair not to take into account the other less striking differences.

We thank the reviewer for pointing this out. We have adjusted the language to make it more clear and to avoid exaggerated claims, as follows:

Cell-DINO outperforms both supervised and SSL approaches by a substantial margin over SimCLR and the supervised approach with only 1% of the available annotations.

- An additional word should be said about the comparison between Supervised and CellDINO in the HPA SC - Protein context where both model F1 scores seem to meet at the 100% sample usage.

We added the following clarifications to the main text:

In all cases at 100% of labeled sample usage, the supervised model has access to all the annotated data to tune all of its parameters. In contrast, Cell-DINO only trains the classification head with supervision and the rest of the backbone parameters are pre-trained with self-supervision and then frozen (no fine-tuning). This large difference in supervised parameters would give an advantage to the supervised approach. However, the Cell-DINO models meet and sometimes surpass the performance of supervised models, highlighting the benefits of self-supervision. These include data efficiency that maximizes learning without labels first, and prevention of potential overfitting issues that facilitates generalization.

- Finally it's unclear why the HPA SC dataset is more challenging. Considering the results in Fig3 results, I would have expected the Protein localisation studies to be the most challenging independently from the FoV/SC status. Could the authors explain further their assessment?

We added the clarification to the main text as follows:

Note that at the FoV level, a protein localization class is considered valid if at least one cell in the FoV has the localization of interest. It is usually the case that multiple cells exhibit the localization, resulting in more evidence to select the correct class. Protein localization at the single cell level is generally more challenging because the pattern needs to be recognized in one cell or the correct classification will be missed. In addition, single-cell training images are not fully annotated, but rather have a weak label inherited from the FoV. Thus, a cell may have a weak positive label assigned when it is actually a negative example. This inconsistency makes it harder to learn from single-cell annotations, and this was by design in the original single cell Kaggle competition because of the difficulty and the challenges of annotating single cells manually [27].

Cell-DINO excels at unbiased profiling of cellular morphology
- Table 2: Cell-DINO+ is used in this table again, I'm not sure to understand why it is only used here and not in the other comparisons?

We thank the reviewer for pointing this out. To keep the results clean and consistent, we have removed the references to Cell-DINO+ from Table 2. As explained before, we reported these results as a potential way of improving results, but from the scientific point of view, comparing against it may obscure the most important reasons for why the Cell-DINO approach is superior (it's not just the initialization). We present a systematic study that separates one aspect of the method at a time to convince ourselves and the community that the approach yields better performance under various circumstances, not just when it has an advantage with better initialization.

- Table 2: If Cell-DINO+ is excluded from this table then Cell-DINO is indeed showing the best results in 3 out 4 contexts, however it seems to only be compared to a single other model. The text is stating "other approaches", would it be possible to add more models here?

We thank the reviewer for this suggestion. In the previous version of the table we only compared Cell-DINO with SimCLR. We have expanded the table with new experiments using the two pre-trained models evaluated in previous sections. The results are as follows:

Table 3. **Nearest neighbors search evaluation.** *F1-scores obtained with the HPA-FoV and HPA-SC datasets on the protein localization (PL) classification and cell-line (CL) prediction tasks.*

Field-of-View (FoV)				
Method	***SSL Type***	***Supervision***	***PL***	***CL***
OpenPhenom [26]	*Reconstructive*	-	*11.5*	*39.3*
SimCLR	*Contrastive*	-	*34.8*	***87.6***
SubCell [18]	*Reconstructive*	*Protein ID*	*39.0*	*62.3*
Cell-DINO	*Self-distilation*	-	***76.8***	*84.4*
Single Cell (SC)				
Method	***SSL Type***	***Supervision***	***PL***	***CL***
OpenPhenom [26]	*Reconstructive*	-	*17.0*	*50.1*
SimCLR	*Contrastive*	-	*77.4*	*82.8*
SubCell [18]	*Reconstructive*	*Protein ID*	*78.2*	*79.9*
Cell-DINO	*Self-distilation*	-	***88.5***	***97.4***

- Fig7.: the images shown are not sufficient to provide a good example of the expected spatial or intensity heterogeneity. The images are too small to conclude and the arrows are not clearly pointing to specific cells or part of cells. Please consider adding a supplementary figure for these cells and an explanation of the expected results.

We have added higher resolution images to the figure and full resolution images to the supplementary material. To clarify, these annotations come from the HPA website, and we expect Cell-DINO features to reflect the expert annotations to some degree. The figure illustrates this agreement qualitatively.

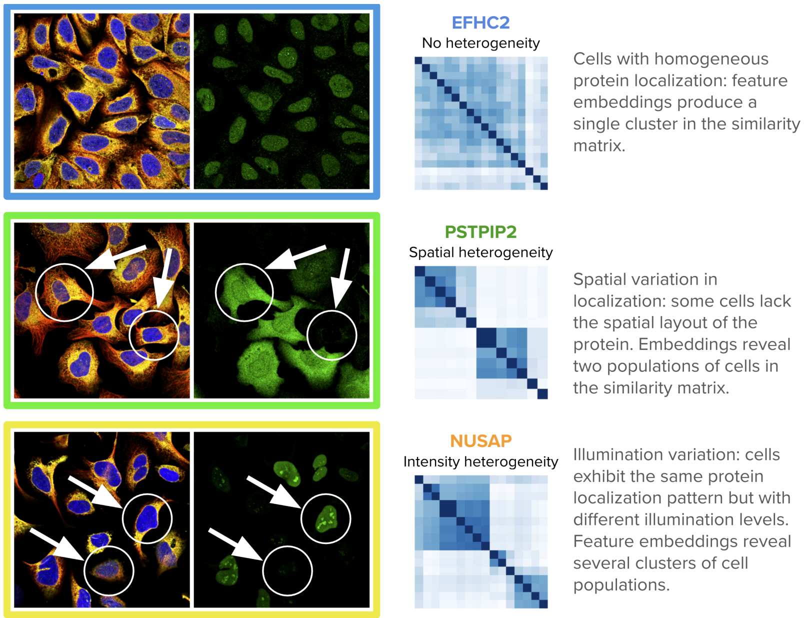

*Figure S3. **Large version of data in Figure 8 from main text.** Single-cell heterogeneity analysis on the HPA-SC dataset. Only U2OS cells were selected for this analysis to measure morphological differences for three proteins EFHC2, PSTPIP2, and NUSAP, which have been annotated as exhibiting no heterogeneity, spatial heterogeneity, and intensity heterogeneity, respectively. The matrices display similarity of single cells from images labeled for the corresponding proteins using Cell-DINO embeddings. Color images illustrate the composite multi-channel visualization, and green images represent the corresponding protein channel. The arrows point to cells that differ in their protein localization activity, explaining the heterogeneity patterns observed.*

Conclusion

I fully agree with the authors that Cell-DINO is an effective strategy with the great benefits of creating less biased embeddings and requiring less annotated data. However, I'm not sure the general conclusion that Cell-DINO ”outperforms supervised approaches and other self-supervised strategies” is supported enough by their comparisons considering that only a couple of these approaches/strategies were compared and that displayed results are only showing a better efficiency in some cases compared to the other models (see Fig 3-4-5, Table 1-2), a more nuanced phrasing may be more adapted or adding additional models could also further strengthen their conclusion.

We appreciate that the reviewer recognizes the effectiveness and the benefits of the proposed Cell-DINO approach, and we thank the reviewer for the suggestion to moderate the language in the conclusions section. We have rewritten the section as a ”Discussion” section, and focus on highlighting the findings supported by our results. In addition, we added discussions about open problems not addressed in this work, including larger scale experiments with JUMP-CP, training channel-agnostic models, creating user-friendly in-

terfaces, and batch effects related issues. We aspire to report relevant cutting-edge research in a transparent and objective way, and we believe the new version is more open and objective.

Methods

- Cell Painting: Would it be interesting to test for lower drug concentration with expected milder effects? Considering the good performance of Cell-DINO in creating unbiased and accurate embeddings, I would be curious to see if it outperforms the other methods when differences are less pronounced.

We have conducted an evaluation with lower drug concentrations and report the results in the supplementary material, as follows:

Table S4. Dose effect evaluation. Comparison of model performance in the mechanism of action (MoA) classification task using the LINCS Cell Painting dataset. The reported values are the area under the precision-recall curve, following the evaluation protocol in [38].

Method	*Low dose*	*Medium dose*	*Maximum dose*
OpenPhenom [26]	*4.97*	*5.92*	*6.40*
CellProfiler	*5.17*	*6.00*	*6.70*
CP-CNN [28]	***5.58***	*6.49*	*7.32*
SubCell [18]	*5.49*	*6.67*	*7.65*
Cell-DINO	*5.47*	***6.70***	***7.67***

The results in Table S4 show how the ability of all models to distinguish MoAs from image-based profiles decreases with dose. The results are the average of ten runs for each model-dose combination. The phenotype of drugs activates at characteristic dosages, which are usually identified using drug-response curves. Our analysis is consistent with the idea that some phenotypes are either not activated or not strong enough to be detected with image analysis. However, our Cell-DINO approach remains robust and performs well as the dosage decreases, outperforming other methods at medium and maximum doses and remaining competitive at low doses.

- In the methods: When describing the Cell-DINO algorithm, it's unclear how the DINOv2 algorithm differs from Cell-DINO.

Thank you for pointing this out. We have clarified this by removing ambiguous language and emphasizing that Cell-DINO is the name we use for models trained with DINOv2 using cellular images. In other words, the algorithm is the same, but in a different domain. This is now explicitly stated in the Methods section, as follows:

At the core, Cell-DINO is the same self-supervised strategy as DINOv2, which is based on the DINO (self-DIstillation with NO labels) algorithm. The adaptations needed to make DINOv2 work with images of cells are primarily intended to accommodate different numbers of channels in the architecture, and to pre-process pixel data according to best practices in microscopy. The self-supervised training algorithm used to create Cell-DINO models does not change with respect to the DINOv2 algorithm [30].

Minor notes: - An effort should be made to harmonize the data set namings: CL/Cell Lines, Protein localization/Prot loc./Protein/PL/Protein loc./protein-localization, HPA-SC/HPA single cell, etc... - Figure 3 is very complex to read and should be reorganized to support the author's hypothesis better - In general, figures are complex to read and should be improved.

We thank the reviewer for these suggestions. We have standardized the naming conventions in figures and tables with the acronym PL and CL. In addition, we have expanded the captions of figures to be more descriptive, and made sure that the resolution is sufficient for reading the presented results.

Reviewer 3

1. Clarify Metric in Figure 3 Caption.
It would be helpful to explicitly state in the Figure 3 caption that the metric being plotted is the F1-score, to avoid ambiguity for readers unfamiliar with the evaluation protocol.

We have expanded the description of Figure 3 in the caption, adding the suggested clarification.

All reported results are the F1-score of the corresponding classification task.

2. Legend Label in Figure 4.
In Figure 4, the label "DINO: 0.55" (blue) could be renamed to "Cell-DINO: 0.55" for consistency with terminology used throughout the paper and to distinguish it clearly from DINOv2 or other models. Also, there is a minor typo in "Kaggle: 0:54" — this should be corrected to "Kaggle: 0.54".

Thank you for pointing this out! We have corrected the labels in Figure 4 accordingly.

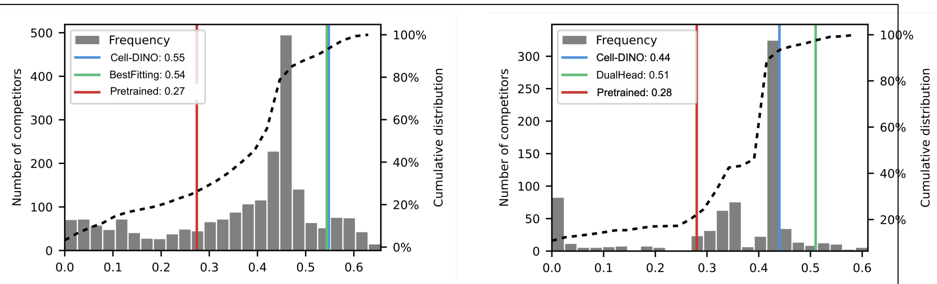

3. Clarify Acronyms in Table 1.
In the Table 1 caption, the acronym AUPRC could be spelled out as "Area Under the Precision-Recall Curve", especially since it is used only for the Cell Painting tasks listed at the bottom of the table.

Thank you for this suggestion, we have updated the caption as follows:

Results using Cell Painting images on the LINCS mechanism-of-action (MoA) prediction task using the Area Under the Precision-Recall Curve (AUPRC) metric [37], and on treatment classification using a subset of the JUMP-CP dataset (accuracy) [4].

4. Repeat Augmentation Definitions in Table 3 Caption.
Although RB (random brightness), RC (random contrast), and RCD (random channel dropout) are defined earlier in the text, repeating these definitions in the Table 3 caption would improve clarity and make the table more self-contained for standalone reading.

We appreciate the suggestion to make the text more self-contained. The caption has been udpated with the following:

RB: random brightness, RC: random contrast, and RCD: random channel dropping.

5. Add a Dedicated Section on Methodological Differences with DINOv2 The paper would benefit from a clearer, dedicated comparison between Cell-DINO and DINOv2. As a reader, it was initially unclear what aspects of the approach are novel or domain-specific (e.g., input channels, augmentations, data modality) versus what is inherited from DINOv2. The section “Cell-DINO Algorithm” helps but could be split into two subsections—one briefly describing the base DINOv2 method, and one clearly listing the adaptations introduced in Cell-DINO. An explicit breakdown would help highlight the novelty of the contributions.

We thank the reviewer for this suggestion. We have reorganized the description of DINOv2 and clarified what are the aspects that make Cell-DINO different in implementation. In short, the core algorithm is the same, and the adaptations introduced are added to support images with different numbers of channels and augmentations consistent with microscopy.

Cell-DINO algorithm

At the core, Cell-DINO is the same self-supervised strategy as DINOv2, which is based on the DINO (self-DIstillation with NO labels) algorithm. The adaptations needed to make DINOv2 work with images of cells are primarily intended to accommodate different numbers of channels in the architecture, and to pre-process pixel data according to best practices in microscopy. The self-supervised training algorithm used to create Cell-DINO models does not change with respect to the DINOv2 algorithm [30]. Here, we describe the components of the base DINOv2 algorithm, and then highlight the main adaptations introduced for Cell-DINO.

DINOv2. *DINO is composed of two networks, student and teacher, that share their architecture. Both networks receive an image and output a feature vector that is then passed on to a projection network that classifies the feature vectors to a logit vector of 65,536 values. The weights of the student network are trained with the cross entropy loss between the outputs of the student and teacher networks. The weights of the teacher network are updated using the exponential moving average of the student network. There are additional losses as part of DINOv2, a Koleo loss and an Ibot loss. After training, the projection head is removed from the feature extractor networks, and the trained teacher network is preserved for feature extraction. The images that the two networks receive are views of the same sample, and augmentations are used to introduce low-level signal alterations that prevent encoding trivial features. Thus, as the two networks are trained to output a similar feature vector for all views of the same image, the feature extractor learns to be invariant to the changes between the views, and encode only the non-changing aspects of the image.*

Adaptations for Cell-DINO. *Differences in channel number and image content with respect to natural images require to adjust augmentations for biological images. This is done primarily to make sure DINO learns relevant visual features for this type of data. The number of channels of the ViT networks is adjusted independently for each dataset to 4 and 5 channels in the HPA and Cell Painting datasets, respectively. We used cross-validation with a subset of 5,000 FoV images from the HPA dataset to test the effect of introducing different augmentations and trained DINO models for 100 epochs. A linear classification model was then trained on the resulting learned features to evaluate performance. We found the following augmentations to be unnecessary for images of cells: Gaussian blurring, solarization, and greyscale transformations. In addition, we found several other augmentations to be useful: randomly rescaling the intensity of the protein channel (in HPA), dropping the content of a random channel by zeroing out all pixels, replacing color jitter with random brightness and contrast changes to each channel individually. These findings are consistent with a previously reported systematic study of image augmentations for self-supervised learning [23].*

6. Clarify and Justify the Term "Unbiased Embeddings".

The term "unbiased embeddings" is used extensively but is not formally defined or empirically quantified. Specifically: There is no explicit test of invariance to known sources of bias in microscopy, such as: -Batch effects -Illumination differences -Image transfor-

mations (e.g., flips, intensity rescaling). The claim of ”unbiasedness” is currently inferred from downstream performance and qualitative visualizations (e.g., UMAPs). A more formal framing or even a simple empirical test of robustness would strengthen this claim and improve interpretability.

We thank the reviewer for pointing this out. In line with suggestions by other reviewers, we have removed the term ”unbiased” from the manuscript. We believe this was confusing and did not convey the main point that we aimed to share with the scientific community. Specifically, we now emphasize the fact that Cell-DINO can learn meaningful representations in a purely self-supervised way, that is, without any labels, auxiliary guidance, or priors from the domain.

> *... our focus is to investigate the properties of Cell-DINO and purely self-supervised strategies to extract meaningful information from biological images.*

We also regret the confusion about ”unbiased” meaning robust to batch effects. While Cell-DINO embeddings are high-quality and useful for downstream analysis, they can be biased by technical variation and still need batch correction. We added a new section to openly discuss this issue and with more details about our observations.

> ***Cell-DINO embeddings encode technical variation***
> *We quantitatively evaluate the difference between using Cell-DINO embeddings with and without batch correction on the MoA prediction task with the LINCS Cell Painting dataset. In this experiment, single-cell embeddings are aggregated hierarchically using the mean of features per field of view, then per well, and finally per treatment. Sphering [1, 28] is used as the batch correction procedure right after per-well data aggregation, and before treatment-level aggregation. The results indicate that the ability to predict MoAs is reduced by 28% without batch correction (Table 4), resulting in a significant degradation of performance. We used the same procedure to measure the impact of not batch-correcting data in feature embeddings learned by other methods. The results in Table 4 confirm that all image-based embeddings exhibit a similar degradation in performance when batch effects are not addressed (see Supplementary Material). In this case, batch effects are a negative bias, but in other cases, they can be a positive bias that overestimates predictive performance. Either way, the best practice is to always test for the presence and impact of batch effects to prevent confounded results. Batch effect correction for imaging data has witnessed remarkable progress recently [33, 3], but it remains an open problem in the general case, and more research is necessary to design effective and robust batch correction algorithms.*

The results are reported in the following table:

Table 4. ***Model Performance Comparison With and Without Batch Correction (B.C.) on the LINCS Cell Painting dataset.*** *The downstream task is MoA classification and the performance metric is PR-AUC. The reported value is the mean of 10 experiments.*

Model	***With B.C.***	***Without B.C.***	***Percent drop***
CP-CNN [28]	*7.32* $\pm$ *0.21*	*5.54* $\pm$ *0.17*	*-24.26%*
OpenPhenom [26]	*6.40* $\pm$ *0.15*	*4.09* $\pm$ *0.25*	*-36.10%*
SubCell [18]	*7.65* $\pm$ *0.17*	*6.18* $\pm$ *0.19*	*-19.23%*
Cell-DINO	*7.67* $\pm$ *0.29*	*5.48* $\pm$ *0.33*	*-28.45%*

7. Deeper Exploration of the Augmentation Space.
While Table 3 offers useful insight into specific augmentations, a more exhaustive exploration of the augmentation space would add significant value. For example, similar to Figure 5 in the SimCLR paper, a systematic study could help identify which augmentations are most beneficial or detrimental for microscopy, and whether they generalize across datasets or phenotyping tasks.

We agree that adjusting the data augmentation pipeline is necessary to make the self-supervised learning algorithms work in biological imaging. We found that the study suggested by the reviewer was previously conducted by Kim et al. [23], and can be found in their Supplementary Figure 1. We report in the Methods section the augmentations that we found useful in our cross-validation experiments, and their findings match our observations in the sense that 'color' augmentations seem to be more important than others. We did not conduct a systematic augmentation study, like they did, because it is computationally expensive and according to our preliminary exploration, we obtain diminishing returns in terms of robustness to batch effects or significant performance improvements, beyond what we already reported. We clarify this in the paper as follows:

We found the following augmentations to be unnecessary for images of cells: Gaussian blurring, solarization, and greyscale transformations. In addition, we found several other augmentations to be useful: randomly rescaling the intensity of the protein channel (in HPA), dropping the content of a random channel by zeroing out all pixels, replacing color jitter with random brightness and contrast changes to each channel individually. These findings are consistent with a previously reported systematic study of image augmentations for self-supervised learning [23].

8. Discussion of Failure Modes of CellDINO + Interpretability of Embeddings.
It would be helpful to include a brief discussion on: Failure modes of Cell-DINO: Are there particular classes (e.g., rare protein localizations or cell lines) where performance degrades? Interpretability of the learned embeddings: Do they encode organelle shape? Texture? Intensity distribution? Even simple saliency maps or patch-level attention heatmaps could offer insight into what the model is focusing on, which is especially important in biomedical settings.

We thank the reviewer for the suggestions. In terms of the failure modes of Cell-DINO, we now report how it is impacted by batch effects and other types of technical variation more clearly. We did not find examples of systematic failure modes specific to Cell-DINO, but observed that there are challenging issues with microscopy images that are still open, including few and noisy labels. We have expanded the interpretability discussions by adding a new section with visualizations as suggested by the reviewer:

Cell-DINO finds meaningful local features

A qualitative evaluation of the patch-level features computed by Cell-DINO reveals that they encode meaningful features of cell morphology. We used PCA analysis to reduce the dimensionality of patch-level features for a subset of U2OS cells in the HPA-SC dataset and visualize the top three principal components (PCs) in the RGB color space (Figure 8A). The resulting visualizations show how different cellular compartments and their morphological properties are encoded in separate PCs. For instance, most of the nucleus variation is encoded in PC-1 (red), while the outer cytoplasm is encoded in PC-2 (green), and the inner cytoplasm is encoded in PC-3 (blue). This local-feature organization explains the ability of Cell-DINO to encode biologically relevant properties in the resulting output embeddings.

In addition to general cellular structure, patch-level features also encode fine-grain, subtle morphological patterns. We evaluated the ability of patch-level features to match more specific morphology across images by computing the cosine similarity between one token in a query image and all tokens in a reference image. The results in Figure 8B show that a token from the nucleus in a query cell with protein localization in the nucleoplasm matches strongly and specifically to tokens in the nucleus of a reference cell with the same protein localization. In a reference cell with no protein localization in the nucleoplasm, the features still match to the nucleus tokens preferentially, but with less intensity. This indicates that the features encode sufficient information to distinguish general cellular structure as well as their specific and meaningful biological variations.

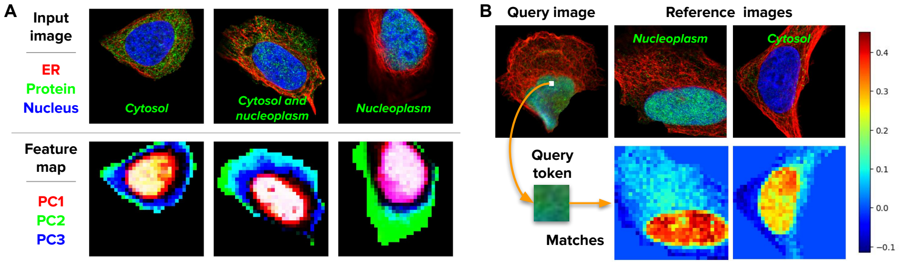

Figure 8. **Patch-level feature maps obtained with Cell-DINO .** *A) Example images and feature map visualizations obtained using principal component analysis (PCA) on patch-level features after masking the background. The first three principal components (PCs) are mapped to the RGB color space. B) Matching patch-level features across images using cosine similarity. A query token is selected from the input image and the cosine similarity is calculated against all patches of reference images.*

References

[1] D Michael Ando, Cory Y McLean, and Marc Berndl. Improving phenotypic measurements in high-content imaging screens. *BioRxiv*, page 161422, 2017.

[2] Anwai Archit, Luca Freckmann, Sushmita Nair, Nabeel Khalid, Paul Hilt, Vikas Rajashekar, Marei Freitag, Carolin Teuber, Melanie Spitzner, Constanza Tapia Contreras, et al. Segment anything for microscopy. *Nature Methods*, 22(3):579–591, 2025.

[3] John Arevalo, Ellen Su, Jessica D Ewald, Robert Van Dijk, Anne E Carpenter, and Shantanu Singh. Evaluating batch correction methods for image-based cell profiling. *Nature Communications*, 15(1):6516, 2024.

[4] Yujia Bao, Srinivasan Sivanandan, and Theofanis Karaletsos. Channel vision transformers: An image is worth c × 16 × 16 words. In *International Conference on Learning Representations*, 2024.

[5] Mark-Anthony Bray, Sigrun M. Gustafsdottir, Mohammad H. Rohban, Shantanu Singh, Vebjorn Ljosa, Katherine L. Sokolnicki, Joshua A. Bittker, et al. A dataset of images and morphological profiles of 30 000 small-molecule treatments using the cell painting assay. *GigaScience*, 6(12), 2017.

[6] Juan C Caicedo, John Arevalo, Federica Piccioni, Mark-Anthony Bray, Cathy L Hartland, Xiaoyun Wu, Angela N Brooks, Alice H Berger, Jesse S Boehm, Anne E Carpenter, et al. Cell painting predicts impact of lung cancer variants. *Molecular biology of the cell*, 33(6):ar49, 2022.

[7] Juan C Caicedo, Allen Goodman, Kyle W Karhohs, Beth A Cimini, Jeanelle Ackerman, Marzieh Haghighi, CherKeng Heng, Tim Becker, Minh Doan, Claire McQuin, et al. Nucleus segmentation across imaging experiments: the 2018 data science bowl. *Nature methods*, 16(12):1247–1253, 2019.

[8] Mathilde Caron, Hugo Touvron, Ishan Misra, Hervé Jegou, Julien Mairal, Piotr Bojanowski, and Armand Joulin. Emerging properties in self-supervised vision transformers. In *Proceedings of the IEEE/CVF International Conference on Computer Vision*, 2021.

[9] Srinivas Niranj Chandrasekaran, Beth A Cimini, Amy Goodale, Lisa Miller, Maria Kost-Alimova, Nasim Jamali, John G Doench, Briana Fritchman, Adam Skepner, Michelle Melanson, et al. Three million images and morphological profiles of cells treated with matched chemical and genetic perturbations. *Nature Methods*, 21(6):1114–1121, 2024.

[10] T. Chen, S. Kornblith, M. Norouzi, and G. Hinton. A simple framework for contrastive learning of visual representations. *Proceedings of the 37th International Conference on Machine Learning*, 2020.

[11] Ting Chen, Simon Kornblith, Kevin Swersky, Mohammad Norouzi, and Geoffrey E Hinton. Big self-supervised models are strong semi-supervised learners. *Advances in neural information processing systems*, 33:22243–22255, 2020.

[12] Xinlei Chen and Kaiming He. Exploring simple siamese representation learning. In *Proceedings of the IEEE/CVF conference on computer vision and pattern recognition*, pages 15750–15758, 2021.

[13] Alice V De Lorenci, Seung Eun Yi, Théo Moutakanni, Piotr Bojanowski, Camille Couprie, Juan C Caicedo, and Wolfgang Maximilian Anton Pernice. Scaling channel-adaptive self-supervised learning. *Transactions on Machine Learning Research*, 2025.

[14] Alexey Dosovitskiy, Lucas Beyer, Alexander Kolesnikov, Dirk Weissenborn, Xiaohua Zhai, Thomas Unterthiner, Mostafa Dehghani, Matthias Minderer, Georg Heigold, Sylvain Gelly, Jakob Uszkoreit, and Neil Houlsby. An image is worth 16x16 words: Transformers for image recognition at scale. In *International Conference on Learning Representations*, 2021.

[15] Marta M Fay, Oren Kraus, Mason Victors, Lakshmanan Arumugam, Kamal Vuggumudi, John Urbanik, Kyle Hansen, Safiye Celik, Nico Cernek, Ganesh Jagannathan, et al. Rxrx3: Phenomics map of biology. *Biorxiv*, pages 2023–02, 2023.

[16] Noah F Greenwald, Geneva Miller, Erick Moen, Alex Kong, Adam Kagel, Thomas Dougherty, Christine Camacho Fullaway, Brianna J McIntosh, Ke Xuan Leow, Morgan Sarah Schwartz, et al. Whole-cell segmentation of tissue images with human-level performance using large-scale data annotation and deep learning. *Nature biotechnology*, 40(4):555–565, 2022.

[17] Jean-Bastien Grill, Florian Strub, Florent Altché, Corentin Tallec, Pierre Richemond, Elena Buchatskaya, Carl Doersch, Bernardo Avila Pires, Zhaohan Guo, Mohammad Gheshlaghi Azar, et al. Bootstrap your own latent-a new approach to self-supervised learning. *Advances in neural information processing systems*, 33:21271–21284, 2020.

[18] Ankit Gupta, Zoe Wefers, Konstantin Kahnert, Jan N Hansen, William D Leineweber, Anthony Cesnik, Dan Lu, Ulrika Axelsson, Frederic Ballllosera Navarro, Theofanis Karaletsos, et al. Subcell: Vision foundation models for microscopy capture single-cell biology. *bioRxiv*, pages 2024–12, 2024.

[19] Sigrun M Gustafsdottir, Vebjorn Ljosa, Katherine L Sokolnicki, J Anthony Wilson, Deepika Walpita, Melissa M Kemp, Kathleen Petri Seiler, Hyman A Carrel, Todd R Golub, Stuart L Schreiber, et al. Multiplex cytological profiling assay to measure diverse cellular states. *PloS one*, 8(12):e80999, 2013.

[20] Kaiming He, Xinlei Chen, Saining Xie, Yanghao Li, Piotr Dollár, and Ross Girshick. Masked autoencoders are scalable vision learners. In *Proceedings of the IEEE/CVF Conference on Computer Vision and Pattern Recognition*, 2022.

[21] Kaiming He, Haoqi Fan, Yuxin Wu, Saining Xie, and Ross Girshick. Momentum contrast for unsupervised visual representation learning. In *Proceedings of the IEEE/CVF conference on computer vision and pattern recognition*, pages 9729–9738, 2020.

[22] R Hollandi, A Szkalisity, T Toth, E Tasnadi, C Molnar, B Mathe, I Grexa, J Molnar, A Balind, M Gorbe, et al. nucleaizer: a parameter-free deep learning framework for nucleus segmentation using image style transfer. cell syst. 10, 453–458. e6, 2020.

[23] Vladislav Kim, Nikolaos Adaloglou, Marc Osterland, Flavio M Morelli, Marah Halawa, Tim König, David Gnutt, and Paula A Marin Zapata. Self-supervision advances morphological profiling by unlocking powerful image representations. *Scientific Reports*, 15(1):4876, 2025.

[24] Alexander Kirillov, Eric Mintun, Nikhila Ravi, Hanzi Mao, Chloe Rolland, Laura Gustafson, Tete Xiao, Spencer Whitehead, Alexander C Berg, Wan-Yen Lo, et al. Segment anything. In *Proceedings of the IEEE/CVF International Conference on Computer Vision*, pages 4015–4026, 2023.

[25] I. Korsunsky et al. Fast, sensitive and accurate integration of single-cell data with Harmony. *Nat. Methods*, 16, 2019.

[26] Oren Kraus, Kian Kenyon-Dean, Saber Saberian, Maryam Fallah, Peter McLean, Jess Leung, Vasudev Sharma, Ayla Khan, Jia Balakrishnan, Safiye Celik, et al. Masked autoencoders for microscopy are scalable learners of cellular biology. In *Proceedings of the IEEE/CVF Conference on Computer Vision and Pattern Recognition*, 2024.

[27] Trang Le, Casper F Winsnes, Ulrika Axelsson, Hao Xu, Jayasankar Mohanakrishnan Kaimal, Diana Mahdessian, Shubin Dai, Ilya S Makarov, Vladislav Ostankovich, Yang Xu, et al. Analysis of the human protein atlas weakly supervised single-cell classification competition. *Nature methods*, 19(10):1221–1229, 2022.

[28] Nikita Moshkov, Michael Bornholdt, Santiago Benoit, Matthew Smith, Claire McQuin, Allen Goodman, Rebecca A Senft, Yu Han, Mehrtash Babadi, Peter Horvath, et al. Learning representations for image-based profiling of perturbations. *Nature communications*, 15(1):1594, 2024.

[29] Théo Moutakanni, Maxime Oquab, Marc Szafraniec, Maria Vakalopoulou, and Piotr Bojanowski. You don't need data-augmentation in self-supervised learning. *arXiv preprint arXiv:2406.09294*, 2024.

[30] Maxime Oquab, Timothée Darcet, Théo Moutakanni, Huy Vo, Marc Szafraniec, Vasil Khalidov, Pierre Fernandez, Daniel Haziza, Francisco Massa, Alaaeldin El-Nouby, et al. Dinov2: Learning robust visual features without supervision. *Transactions on Machine Learning Research*, 2023.

[31] Lukas Minus Orre, Mattias Vesterlund, Yanbo Pan, Taner Arslan, Yafeng Zhu, Alejandro Fernandez Woodbridge, Oliver Frings, Erik Fredlund, and Janne Lehtiö. Subcellbarcode: proteome-wide mapping of protein localization and relocalization. *Molecular cell*, 73(1):166–182, 2019.

[32] Wei Ouyang, Casper F Winsnes, Martin Hjelmare, Anthony J Cesnik, Lovisa Åkesson, Hao Xu, Devin P Sullivan, Shubin Dai, Jun Lan, Park Jinmo, et al. Analysis of the human protein atlas image classification competition. *Nature methods*, 16(12):1254–1261, 2019.

[33] Wolfgang M Pernice, Michael Doron, Alex Quach, Aditya Pratapa, Sultan Kenjeyev, Nicholas De Veaux, Michio Hirano, and Juan C Caicedo. Out of distribution generalization via interventional style transfer in single-cell microscopy. In *Proceedings of the IEEE/CVF Conference on Computer Vision and Pattern Recognition*, pages 4326–4335, 2023.

[34] Mohammad Hossein Rohban, Shantanu Singh, Xiaoyun Wu, Julia B Berthet, Mark-Anthony Bray, Yashaswi Shrestha, Xaralabos Varelas, Jesse S Boehm, and Anne E Carpenter. Systematic morphological profiling of human gene and allele function via cell painting. *Elife*, 6:e24060, 2017.

[35] C. Stringer, T. Wang, M. Michaelos, and M. Pachitariu. Cellpose: a generalist algorithm for cellular segmentation. *Nat. Methods*, 18, 2021.

[36] Carsen Stringer and Marius Pachitariu. Transformers do not outperform cellpose. *bioRxiv*, pages 2024–2004, 2024.

[37] G. P. Way et al. *Morphology and gene expression profiling provide complementary information for mapping cell state*. Cell Syst 13, 911–923, 2022.

[38] G.P. Way, T. Natoli, A. Adeboye, L. Litichevskiy, A. Yang, X. Lu, J.C. Caicedo, B.A. Cimini, K. Karhohs, D.J. Logan, M.H. Rohban, M. Kost-Alimova, K. Hartland, M. Bornholdt, S.N. Chandrasekaran, M. Haghighi, E. Weisbart, S. Singh, A. Subramanian, and A.E. Carpenter. Morphology and gene expression profiling provide complementary information for mapping cell state. *Cell Systems*, 13:911–923, 2022.
