# Supplementary material for: Cell-DINO: Self-supervised image-based embeddings for cell fluorescent microscopy
Source: PLoS Comput Biol. 2025 Dec 29;21(12):e1013828. doi: 10.1371/journal.pcbi.1013828 (PMC12826486; doi:10.1371/journal.pcbi.1013828)
Supplement: Attachment — Submitted filename: response_to_reviewers.pdf [file pcbi.1013828.s003.pdf]

Cell-DINO: Self-Supervised Image-based Embeddings for Cell Fluorescent Microscopy: Response to reviews

Dear Editor,

Thank you very much for your invitation to submit a revision of our manuscript. We have revised the materials according to the referees' comments and suggestions. We want to thank the reviewers for the constructive feedback, and suggestions. The reviewers' original comments, and our responses, are listed below.

Journal Requirements

1) Please ensure that the funders and grant numbers match between the Financial Disclosure field and the Funding Information tab in your submission form. Note that the funders must be provided in the same order in both places as well. Currently, the order of this grant number (2348683) does not match in both the places.

We have looked at the funder numbers and organizing in the required order.

2) The file inventory includes multiple files for Figure 4. We would recommend either combining these into a single Figure 4.tiff file with separate internal panels, or renumbering them as individual figures, as we are not able to publish multiple components of a single figure as separate files.

We have combined the components of Figure 4 in a single file.

Note: If the reviewer comments include a recommendation to cite specific previously published works, please review and evaluate these publications to determine whether they are relevant and should be cited. There is no requirement to cite

these works unless the editor has indicated otherwise.

We thank editor for the clarification, and we confirm that we are not adding additional citations in this revision.

Reviewer 1

The authors have addressed all of the concerns I raised, and I believe the manuscript can be published in its current form. I appreciate their thoughtful responses and commend their commitment to open science. This work will be a valuable contribution to the field of bioimage analysis.

We thank the reviewer for their thoughtful comments and suggestions, and we are glad that our responses have been satisfactory. We appreciate that they find our work a valuable contribution to the field.

Reviewer 2

I thank the authors for carefully considering my comments in their revised manuscript. All of my major concerns have been addressed, and I now have only a few minor observations.

We thank the reviewer for the feedback, and we are glad that the responses satisfactorily addressed the major concerns raised before. Here, we address the new observations to ensure reproducibility of our work.

Minor observations: - In the cell-painting-eval/README file: ”feature extraction need to be performed with the DINOv2 implementation provided here (link to be added soon)”. I expect this code will be released and linked as soon as the paper is accepted to make the pipeline fully reproducible.

We thank the reviewer for pointing this out. We are committed to open science and open source software, and we are glad to report that both repositories are now public and accessible to everyone. The repositories are now reported in the Code Availability Section as follows:

Our implementation is an adaptation of DINOv2, which is hosted in the main DINOv2 repository. This implementation also includes code to reproduce the results of the HPA analysis. The code is available in GitHub at `https://github.com/facebookresearch/dinov2/blob/main/docs/README_CELLDINO.md`*. The code necessary to reproduce the Cell Painting results with the LINCS dataset is publicly available in GitHub at:* `https://github.com/CaicedoLab/cell-painting-devbench`*.*

- A variable seems to be misnamed in the file located at cell-painting-evalLINCSrun_all.sh. Line 12 should read ‘echo ”Error: $CONFIG does not exist.”‘ instead of ‘echo ”Error: $File does not exist.”‘ as to correctly point to the problematic file.

We thank the reviewer for reporting this bug. We have fixed the issue in the main repository.

- I tried to follow the conda environment creation protocol and ended with a pip dependency conflicts both on a Windows and an Ubuntu machine. I would encourage the authors to share a yml recipe to streamline the environment creation as they did in the cell-dino-hpa repo.

We have added a new file to the repository to facilitate installation and reproduction of results. The new file is called cp-lincs-eval-environment.yml. It contains all the dependencies used in our experiments to complete the evaluations. We still recommend to use pip and manage the environment manually whenever possible.

- In the cell-dino-hpa code repo, the main title is still showing the old article title (”# Cell-DINO: Unbiased Image-based Embeddings for Cell Fluorescent Microscopy”)

We thank the reviewer for pointing this out. We have updated the name as part of the pull request.

Reviewer 3

1. Naming: I recommend considering renaming Cell-DINO to Cell-DINOv2. This would align better with future iterations (e.g., Cell-DINOv3 if adapted to DINOv3). Alternatively, if the differences between Cell-DINO and DINOv2 are minimal, the work could be presented as an investigation of how well DINOv2 applies to fluorescent microscopy images. This would help with clarity and long-term consistency. (This would of require re-working Figure 3, but it might make it clear to the reader that what you are presenting is not a new method, but rather the same method DinoV2 but investigated with different initializations and pre-trained on microscopy data and not natural images).

We thank the reviewer for suggesting this stylistic variation. First, we emphasize that do not claim to have a new method; this issue was raised in the previous revision, and we thoroughly modified the texto to clarify this in the abstract, the results, and the methods section.

In addition, we clarify that our current plan does not include producing another version of Cell-DINO with the DINOv3 algorithm. The main goal of our work was to demonstrate that the self-distillation approach, as implemented in DINOv2, is an effective way to encode image-based features for biological studies. We also note that DINOv2 is the name given to a pre-trained vision model, whereas Cell-DINO is not a pre-trained, foundation model. Cell-DINO is meant to be the strategy of not using supervision, but rather the DINO family of algorithms to address cell phenotyping problems with imaging. For all these reasons, we decided to keep the current name: Cell-DINO.

2. Abstract: The abstract could be made clearer. Currently, it opens with: ”Accurately quantifying cellular morphology at scale...” This is more of a downstream application. The core contribution is that the method learns representations that can then enable such quantification/classification. Re-framing the abstract to emphasize “representation learning” upfront would sharpen the message for readers.

We respectfully disagree with the reviewer. Our choice of starting with *quantifying cellular morphology* is based on our intention to prioritize the biological application, which is the main motivation and inspiration for this study. As noted by the reviewer and others, we do not introduce a new technical innovation, and representation learning is not the novel aspect of our work. Others have used representation learning for the same problem, including the SubCell and OpenPhenom teams. The application of DINO to cellular morphology problems is the central message of our work. For this reason, we decided to keep the current style in the abstract, which prioritizes computational biology readers.

3. Title: While the representations are indeed learned in a self-supervised manner, the evaluation still requires training a linear classifier on a small set of labeled data. To reflect this more precisely, consider a title such as: “Self-supervised representation learning for fluorescent microscopy with minimal supervised fine-tuning.” This would set accurate expectations for the reader.

We agree with the reviewer that training a linear classifier, even on a small set of labeled data is sometimes required to solve certain tasks. However, we used linear classifiers and fine-tuning only as experimental tools to measure the quality of the representations. The strength of the representations can also be leveraged without explicit supervision, as it is extensively described in the section ”Cell-DINO excels at image-based profiling of cellular morphology”, where nearest-neighbor classifiers are used in the HPA tasks (Table 3), and where other biologically meaningful analysis is described without the use of classifiers at al (e.g., clustering, matching, search, Figure 7).

We believe that the unsupervised case is the most useful of the applications of Cell-

DINO embeddings, because ground truth data for supervised learning is very difficult to collect in biological studies. For these reasons, we cannot modify the title of our study, given that this stylistic change would be unfair with the value of the representations offered by Cell-DINO.

4. Figure 1: (i) I suggest splitting the figure into two individual figures: (A,B) illustrating the method, and (C) showing the datasets. The dataset figure can stand independently from the method. (ii) In the ViT schematic (B), the sequence of tokens should remain consistent (16 in input, 16 in output), and the attention matrix should reflect 16 × 16 to avoid confusion.

We have applied the suggested style change, as follows: (i) we divided Figure 1 in two different figures, one with the schematics of the method and another with the datasets. (ii) For the schematic of the ViT, we now keep 16 patches in the input and the internal self-attention matrix representations. We preserve the graph figure with only six nodes as this is a cartoon representation of the self-attention matrix for illustration purposes only, and it becomes unreadable with all the 16 patches simultaneously.

5. Figure 2: It would be very helpful to show clustering of single cells for protein localization in the HPA dataset before applying Harmony, for readers to fully appreciate the effect of the correction.

We have added the visualization before and after harmony to the supplementary material, in Figure S2. The figure displays protein localization labels as substructure of cell lines. In other words, protein localization is a secondary factor of variation that can be aligned across cell lines using batch effect correction methods, as discussed in the section ”Cell-DINO encodes diverse biological properties of cells”.

6. Figure 3: You say in the caption ”Protein localization blue shades and Cell line green shades”, but in the legend on the bottom right it looks the other way around (blue for cell line, green for protein localization). Please clarify.

We thank the reviewer for pointing this out. We have fixed the reference. Protein localization is green and cell line is blue.

7. Grammar and Style Corrections: (i) Author Summary: remove the space in “Cell-DINO ,” → “Cell-DINO,” (ii) Introduction: ¿”because the goal is to uncover the effect of such perturbations.” –¿ reference missing and replaced by question mark currently. (iii) Figure 3: “Worse” → “Worst”. (iv) Figure 3 caption: “All reported results are the F1 score” → “F1 scores”. (v) Cell-DINO outperforms alternative self-supervised strategies: ”Mask Autoencoder” should be ”Masked Autoencoder”. (vi) Table 3: ”Self-distilation” should be ”Self-distillation”. (vii) Figure 4: ”BestFitting” should be ”bestfitting” in the legend. (viii) Cell-DINO excels at image-based profiling of cellular morphology: ¿”We find that Cell-DINOembeddings ...” -¿ add a space after Cell-DINO

We thank the reviewer for the thoroughness in proofreading the manuscript. We have applied all the suggested changes to the latest version of the manuscript.

8. Overall: I appreciate that the revised version de-emphasizes the claim of “unbiasedness” of the embeddings, which makes the framing more balanced. The writing can be worked upon more. Currently I feel as a reader that the writing can be tightened in several places.

We really appreicate the reviewer’s comments, and we are glad that our revised version has successfully addressed all major concerns. We have also proofread the manuscript to find other opportunities to improve clarity and style.
